# Cell-Free Systems Biology: Characterizing Central Metabolism of *Clostridium thermocellum* with a Three-Enzyme Cascade Reaction

**DOI:** 10.1021/acssynbio.4c00405

**Published:** 2024-10-10

**Authors:** S. Bilal Jilani, Markus Alahuhta, Yannick J. Bomble, Daniel G. Olson

**Affiliations:** † Thayer School of Engineering at Dartmouth College, Hanover, New Hampshire 03755, United States; ‡ National Renewable Energy Laboratory, Biosciences Center, Golden, Colorado 80401, United States

**Keywords:** 2,3-butanediol, acetolactate synthase, acetolactate decarboxylase, 2,3-butanediol dehydrogenase, formate dehydrogenase, *Hungateiclostridium*, *Ruminiclostridium*, *Acetivibrio thermocellus*

## Abstract

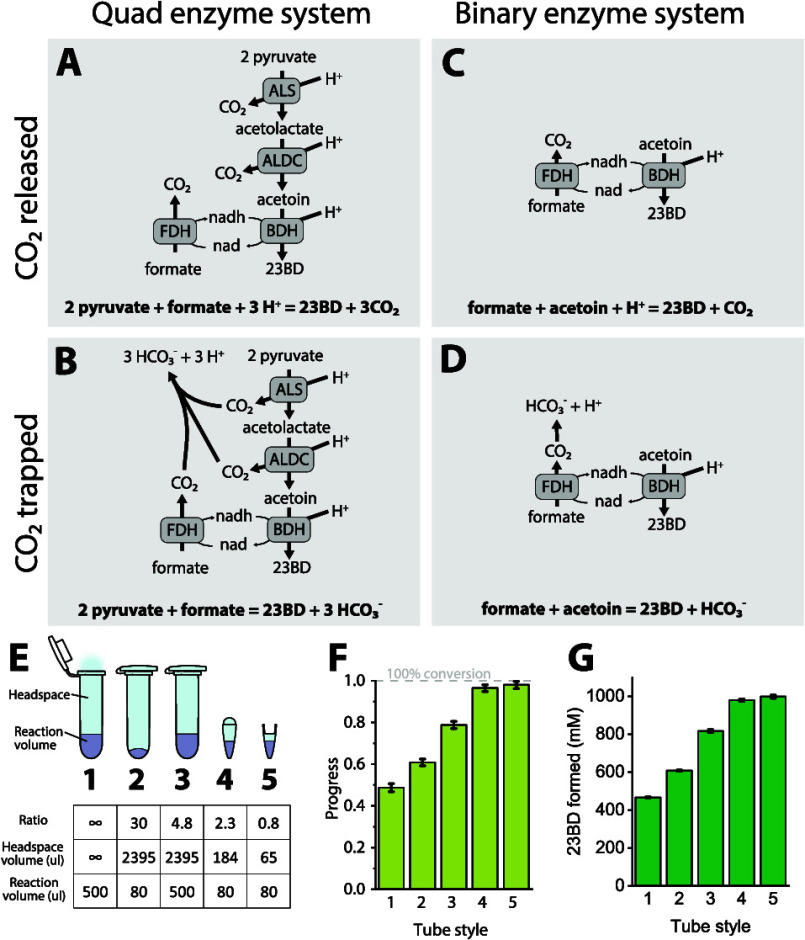

Genetic approaches have been traditionally used to understand microbial metabolism, but this process can be slow in nonmodel organisms due to limited genetic tools. An alternative approach is to study metabolism directly in the cell lysate. This avoids the need for genetic tools and is routinely used to study individual enzymatic reactions but is not generally used to study systems-level properties of metabolism. Here we demonstrate a new approach that we call “cell-free systems biology”, where we use well-characterized enzymes and multienzyme cascades to serve as sources or sinks of intermediate metabolites. This allows us to isolate subnetworks within metabolism and study their systems-level properties. To demonstrate this, we worked with a three-enzyme cascade reaction that converts pyruvate to 2,3-butanediol. Although it has been previously used in cell-free systems, its pH dependence was not well characterized, limiting its utility as a sink for pyruvate. We showed that improved proton accounting allowed better prediction of pH changes and that active pH control allowed 2,3-butanediol titers of up to 2.1 M (189 g/L) from acetoin and 1.6 M (144 g/L) from pyruvate. The improved proton accounting provided a crucial insight that preventing the escape of CO_2_ from the system largely eliminated the need for active pH control, dramatically simplifying our experimental setup. We then used this cascade reaction to understand limits to product formation in *Clostridium thermocellum*, an organism with potential applications for cellulosic biofuel production. We showed that the fate of pyruvate is largely controlled by electron availability and that reactions upstream of pyruvate limit overall product formation.

## Introduction

Microbes have a diverse array of metabolic capabilities that can be used for industrial chemical production. In many cases, however, it is necessary to modify the metabolism of these organisms, and this requires understanding how native metabolism functions. Traditionally, the characterization of microbial metabolism and testing metabolic engineering strategies have been approached using the tools of genetics and molecular biology. This approach works well in model organisms but can be difficult to implement in novel isolates or nonmodel organisms with unique physiology or limited genetic tools. Here, we propose a new approach to this problem using cascade reactions to characterize microbial metabolism in cell lysates.

The conversion of cellulose into biofuels such as ethanol is a process that is currently being investigated as means of producing liquid transportation fuels and chemicals with low or negative carbon dioxide (CO_2_) emissions.^1^ Although cellulose is recalcitrant to microbial deconstruction, there is a class of specialist organisms that can solubilize cellulose efficiently. The most well studied of these organisms is *Clostridium thermocellum*.^2^ There has been significant recent progress in engineering this organism for increased ethanol yield,^3,4^ but improvements in titer have remained elusive. A key question that remains is “what factors limit product titer?”

One of the central challenges in studying the metabolism of this organism is that as a cellulose specialist, it consumes a limited range of substrates (i.e., mostly cellulose and its solubilization products such as cellobiose and longer-chain glucans), and all of these substrates enter metabolism via glycolysis. Furthermore, glycolysis is the only pathway for dissimilation of these compounds into metabolic intermediates.^5^ This combination of metabolic features precludes the use of genetic techniques to study metabolism (since many key metabolic genes are essential) and also precludes feeding with intermediate metabolites.

To overcome these problems, we recently developed a cell-free extract reaction (CFER) system to study the metabolism of this organism.^6^ The use of cell lysates gives direct access to the cytoplasm for the addition of intermediate metabolites. However, metabolites are linked in complex metabolic networks, and adding a large concentration of a single metabolite often causes stoichiometric imbalances that completely block metabolism.^6^ To avoid this problem, we realized that we needed to identify enzymatic reactions that could be introduced into our cell lysates that could serve as “sources” or “sinks” of intermediate metabolites. We hypothesized that these reactions would allow us to understand the metabolic network of this organism directly in the cell lysate.

Much of this prior work has focused on the question of understanding how the metabolism functions. In this work, we are interested in asking a slightly different question: what are all of the ways that metabolism *could* function? To do this, we are borrowing a coupled enzyme assay technique from the field of enzymology. In this technique, an enzyme whose activity is difficult to observe can be coupled to another enzyme (or enzyme cascade) to provide a readout that is easier to detect.^7^ Redox enzymes are commonly used as coupling enzymes due to the ease of measuring nicotinamide cofactors (NADH and NADPH) by a spectrophotometer. We have extended this technique by applying it to study pathways in cell lysates. By combining the addition of various metabolites and/or well-characterized enzyme modules, we are able to isolate and perturb metabolic modules within the metabolic network. In this work, we demonstrate a proof-of-concept of this approach, which we call “cell-free systems biology” in *C. thermocellum*, to better understand metabolic control in this organism.

Two key questions we wanted to answer were (1) is ethanol flux primarily limited by glycolysis (sugar to pyruvate) or fermentation pathways (pyruvate to ethanol) and (2) are fermentation pathways controlled primarily by carbon or electron availability. We therefore set out to develop tools to study metabolism in vitro, an approach we call cell-free systems biology. Pyruvate is a key intermediate metabolite that sits at the junctions of glycolysis and fermentation. Therefore, our first step was to develop a reaction that could serve as a sink for pyruvate.

## Results

With the overall goal of better understanding the metabolism of *C. thermocellum*, we set out to develop an orthogonal reaction system that could act as a sink for key metabolic intermediates. We chose to target the intermediate metabolite pyruvate since it sits at the junction of the glycolysis and fermentation pathways in central metabolism. We chose to use 2,3-butanediol (23BD) production as a pyruvate sink because the pathway does not natively exist in*C. thermocellum*, the enzymes in this pathway are all known, all of the steps are thermodynamically favorable, most of the products and reactants are stable and easily measured (with one notable exception of 2-acetolactate), and high titers of 23BD have been reported in the literature for both cell-based^8−10^ and enzyme-based systems.^11,12^

**Figure 1:**
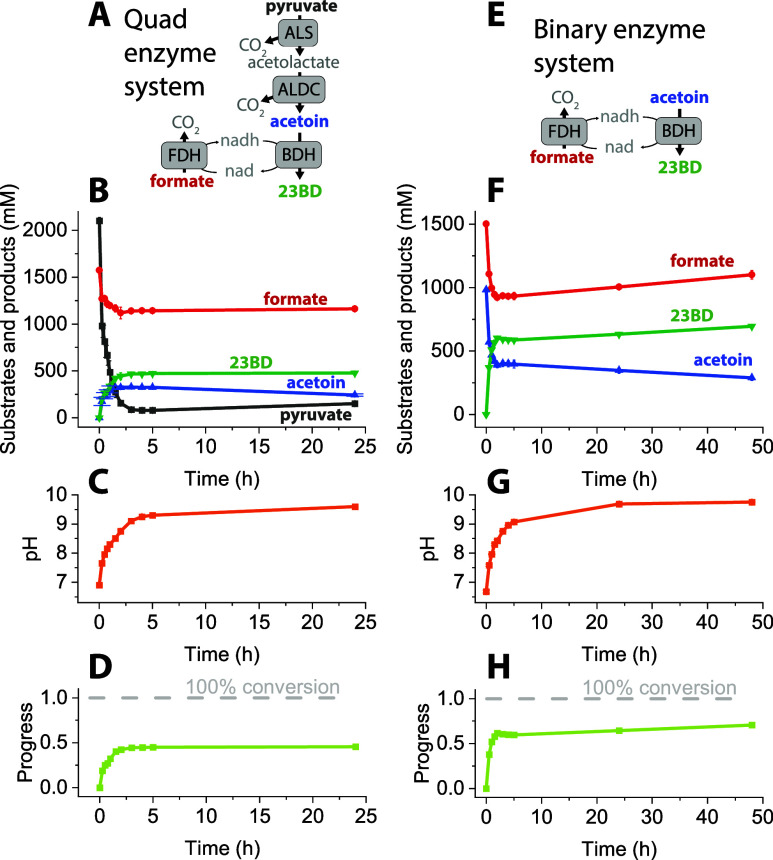
Conversion of pyruvate or acetoin to 2,3-butanediol (23BD) using either a quadruple or binary cascade reaction. Panel A shows the quad system consisting of acetolactate synthase (ALS), acetolactate decarboxylase (ALDC), 23BD dehydrogenase (BDH), and formate dehydrogenase (FDH). Panel B shows the concentration of substrates and products in the quad system, including formate, pyruvate, acetoin, and 23BD. Panel C shows the pH of the quad system. Panel D shows the reaction progress of the quad system. 100% reaction progress would indicate the conversion of 2 mol of pyruvate into 1 mol of 23BD. Panels E–H show similar information as panels A–D, but for the binary enzyme system. Note that in the binary system, 100% reaction progress would indicate the conversion of 1 mol of acetoin into 1 mol of 23BD. Enzyme reactions were performed in a 0.5 mL volume at 37 C. Enzymes were present in the reaction mixture in the following amounts: ALS, 10 μg; ALDC, 75 μg; BDH, 95 μg; and FDH, 400 μg. Error bars represent the standard deviation from the two biological replicates. In some cases, the error bars are smaller than the data markers.

The first step in developing the pyruvate to 23BD pathway as a sink reaction was to demonstrate efficient, high titer, and prolonged conversion in vitro. To achieve this, we used three heterologously purified enzymes—acetolactate synthase (ALS), acetolactate decarboxylase (ALDC), and 2,3-butanediol dehydrogenase (BDH)—which convert pyruvate to 23BD. The last of these, BDH, utilizes NADH as a cofactor which needs to be regenerated for efficient functioning of the pathway. Thus, we introduced a fourth enzyme, formate dehydrogenase (FDH). In the presence of formate, the FDH enzyme regenerates NADH by reduction of NAD^+^ generated by the BDH reaction. We subsequently refer to this as the “quad” enzyme system. This system performs a net conversion described by the equation: 2 pyruvate + formate → 3CO_2_ + 23BD (Figure 1).

### Substrate Conversion Efficiency in the Initial Reaction Setup

In our initial tests, we found that the reaction proceeded rapidly for the first few hours but then slowed down and stopped at only 50% completion (Figure 1, panels B,D). An interesting observation was the rapid increase in the pH of the reaction system from the starting value of 7 to greater than 9 at the end of 24 h (Figure 1, panel C). To better understand the dynamics of the reaction, we simplified our system to just the two enzymes BDH and FDH (i.e., the “binary” enzyme system) (Figure 1, panels E–H). With this simplified system, we observed an increase in the extent of conversion to 70%, but the reaction was still incomplete with a significant amount of substrate remaining. In the binary system, we also observed a steady increase in the pH value of the system from a starting value of 7 to greater than 9 at 24 h and onward (Figure 1, panel G). The importance of monitoring the pH in cell-free systems has been reported in earlier studies.^13−15^ Thus, we next investigated the role of pH on enzyme activity.

### Stability of Enzymes as a Function of Time and pH

**Figure 2:**
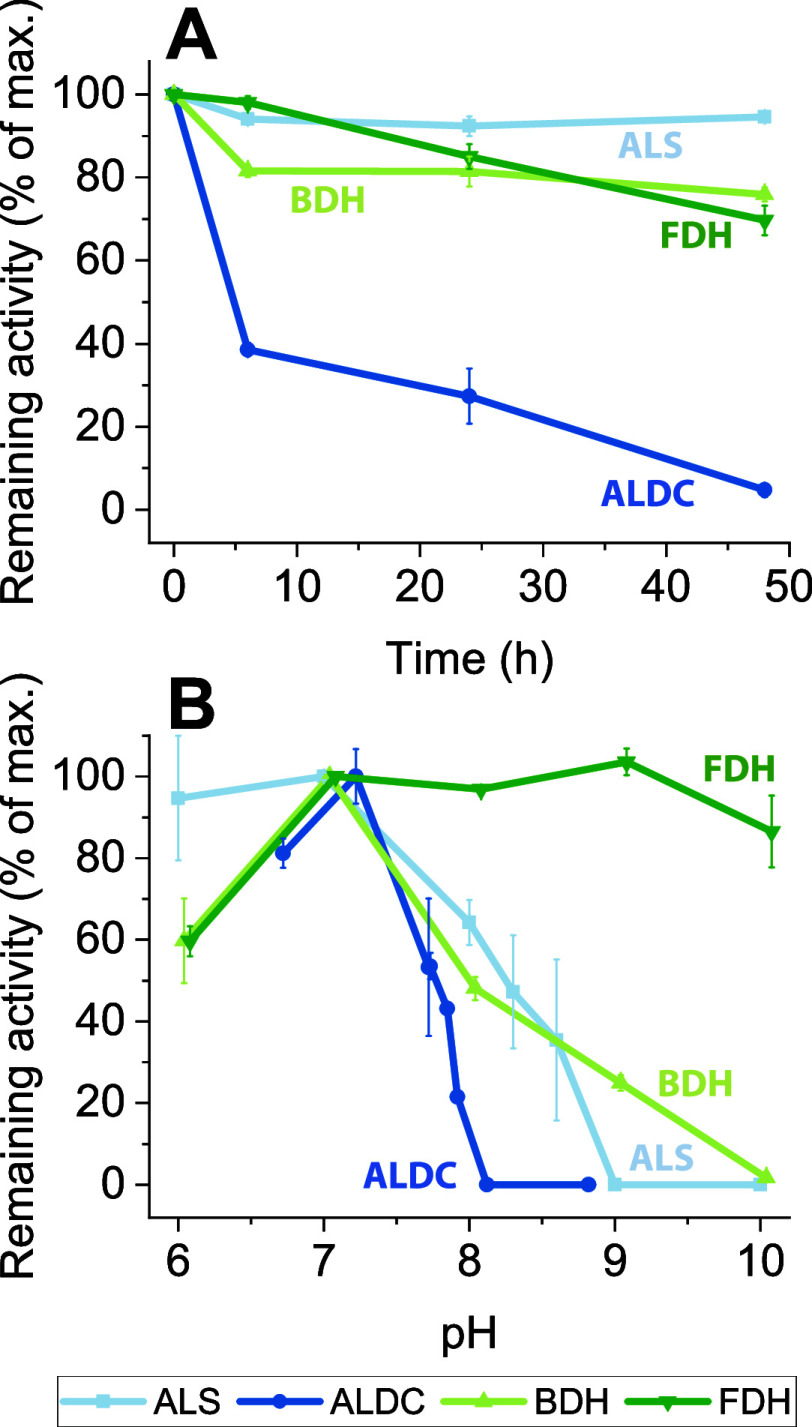
Measurement of the enzyme stability and activity. Panel A shows enzyme activity vs time, normalized to initial activity. Panel B shows initial enzyme activity versus pH, normalized to the maximum activity for each enzyme. Error bars represent one standard deviation from two biological replicates.

Since catalytic activity of the enzymes may be influenced by both prolonged incubation at 37 °C as well as changes in pH values, we next evaluated the performance of all four enzymes as a function of incubation at 37 °C (Figure 2A) and variation in pH (Figure 2B).

We observed that ALS, BDH, and FDH enzymes were resilient to incubation at 37 °C with all three of them maintaining >70% of the activity, as compared to *T* = 0 h, at the end of 48 h. The ALDC enzyme proved to be the most unstable, exhibiting only 39% of the initial activity at 6 h and only 5% by 48 h. As a function of variation in the pH (Figure 2B), we also observed that the ALDC enzyme is the most unstable. At pH value above 8, we were not able to observe any catalytic activity associated with ALDC, while the other three enzymes were >50% catalytically active at similar pH. At pH 9, no activity associated with ALS could be detected. This suggested that increases in pH might explain the low conversion of the substrate in our initial tests (Figure 1). The cessation of catalytic activity of ALS and ALDC at pH 9 and 8, respectively, together with >9 pH value observed in our reaction mixes suggest that enzyme activity could be the limiting factor in efficient conversion of pyruvate or acetoin to 23BD.

### Active pH Control Increases Substrate Conversion

**Figure 3:**
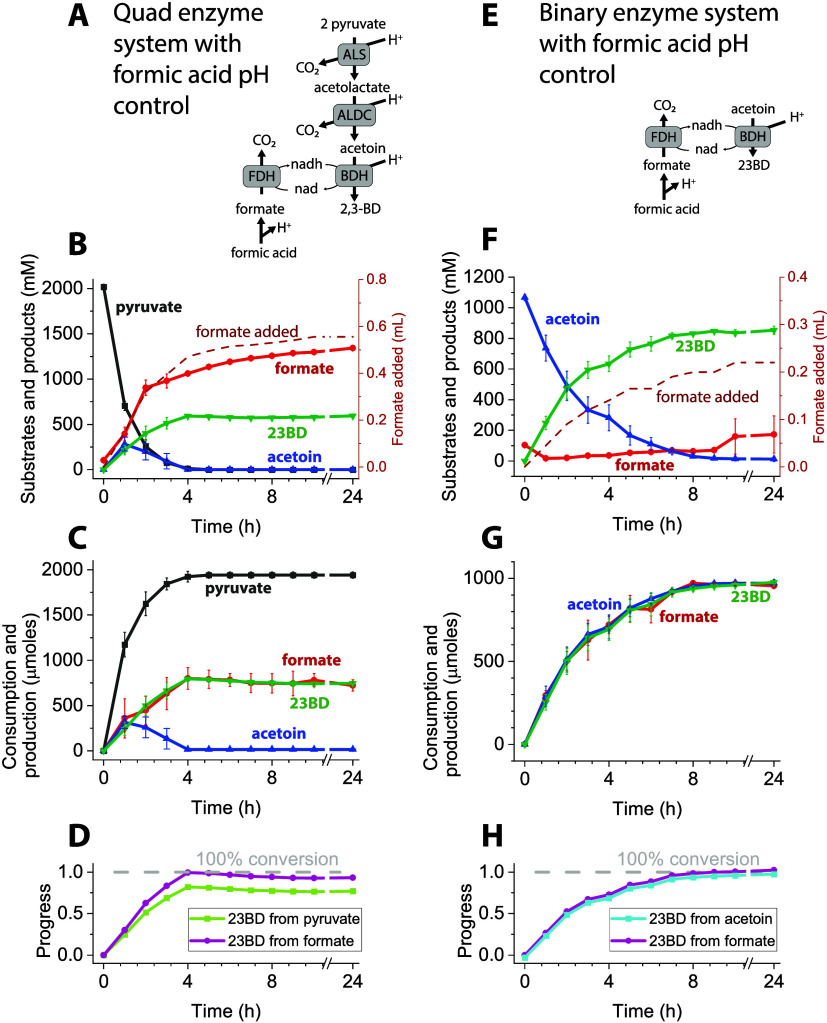
Batch reactions with active pH control. Panel A describes the quad enzyme reaction when the pH is controlled by the addition of formic acid (4.6M). The dashed line showing formate addition corresponds to the right axis. Panel B shows the concentrations of substrates and products during the course of the fermentation. Panel C shows the consumption of substrates (pyruvate and formate) and the production of products (acetoin and 23BD). Here quantities (μmoles) rather than concentrations (mM) are shown to allow the stoichiometry of the reaction to be observed more clearly, without interference from effects related to dilution from the addition of formate. Panel D shows the progress of the reaction, which can be calculated based on either formate conversion (1 mol of 23BD per mole of formate consumed) or pyruvate (1/2 mol of 23BD per mole of pyruvate consumed). Panels E–H show the corresponding data for the binary enzyme system. The reaction volume was 1.0 mL. Concentrations of enzymes were ALS 20 ug/mL; ALDC 150 ug/mL; BDH 190 ug/mL; and FDH 800 ug/mL. Error bars represent one standard deviation from two biological replicates.

We hypothesized that controlling the pH would allow more complete conversion of the substrates into products. To test our hypothesis, we constructed a batch enzyme reactor, where the increase in pH was controlled by the addition of dilute acid. We selected dilute formic acid for pH control since it provides both protons (for pH control) and formate (a substrate for the FDH reaction) (Supporting Figure S3). We observed a much higher efficiency of substrate conversion to product in the pH controlled experimental setup. In the binary system, the acetoin substrate was completely consumed and converted to a product at ∼100% of the theoretical maximum yield. Formate addition closely matched 23BD production, as expected from the balanced proton stoichiometry (one proton produced by dissociation of formic acid was consumed by the BDH reaction) (Figure 3).

In the quad system, the pyruvate was completely consumed and mostly converted to 23BD. The maximum efficiency was around 99% of the theoretical maximum (based on formate conversion) or 82% (based on pyruvate conversion). Although we would have expected these values to be identical, it is possible that some of the pyruvate is converted to 2-acetolactate, which we did not measure. One problem with the quad system is that the proton stoichiometry is not balanced. For each mole of 23BD produced, 3 protons are required, but only one mol of formate is required. Since our formic acid addition was based on pH control (i.e., proton demand), more formic acid was added, compared to what was consumed for cofactor (NADH) recycling by the FDH reaction, and this explains the increase in formic acid over time (Figure 3). Although in theory feeding a mixture of pyruvic and formic acid could allow for an exact proton balance, we found that these mixtures generally did not allow for high conversion, possibly due to system stability issues (Supporting Figure S4).

### Testing Limits of Substrate Conversion to 23BD

**Figure 4:**
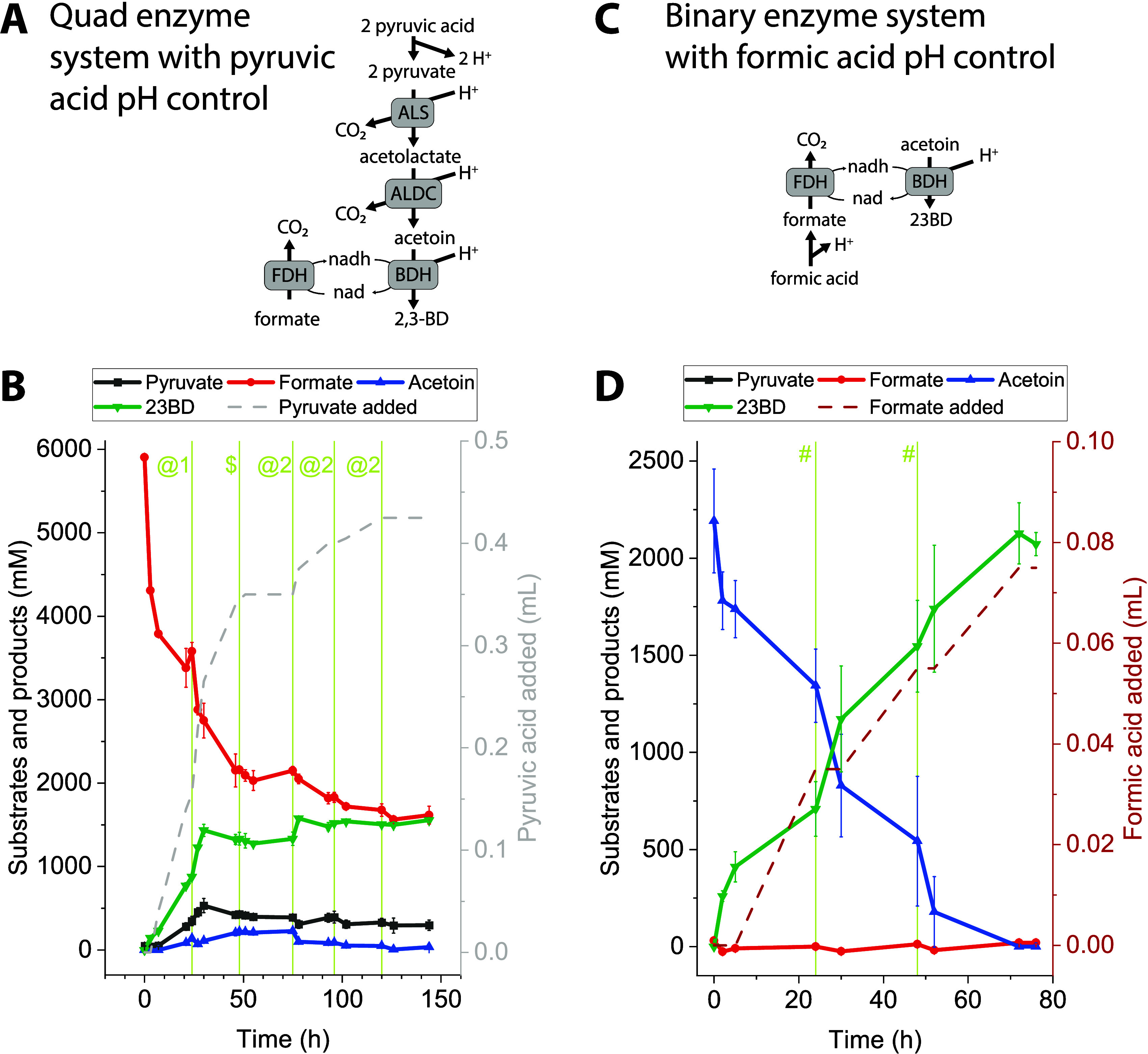
Identifying limitations of 23BD production. Panel A describes the quad enzyme reaction system, where the pH is controlled by the addition of pyruvic acid (14.1 M). Panel B shows the concentration of substrates and products. Addition of pyruvic acid is shown with a dashed gray line that corresponds to the right axis. Green vertical lines indicate the addition of additional reaction components. The @ symbol indicates addition of all four enzymes and NADH. At the @1 symbol, 1 mM NADH; 40 ug ALS; 150 ug ALDC; 190 ug BDH; and 800 ug FDH were added. At the @2 symbol, 1 mM NADH; 20 ug ALS; 150 ug ALDC; 190 ug BDH; and 800 ug FDH were added. The $ symbol indicates the addition of 1 mM NADH. Panel C describes the binary enzyme reaction system, where the pH is controlled by the addition of formic acid (23.3 M). Green vertical lines indicate the addition of additional reaction components. Panel D shows the concentration of substrates and products. The reaction volume was 0.8 mL. The dashed red line shows the addition of formic acid, which corresponds to the right axis. Initial concentrations of enzymes were ALS 20 ug/mL; ALDC 150 ug/mL; BDH 190 ug/mL; and FDH 800 ug/mL. Error bars represent one standard deviation from two biological replicates. The # symbol indicates addition of 1 mM NADH and 190 ug of BDH.

Having addressed limitations due to variations in pH, we were curious about what other factors might limit titer at higher substrate concentrations. For the quad system, we switched from formic acid to pyruvic acid to control the pH. The reason for this is related to proton stoichiometry. Since two moles of pyruvate are consumed per mole of 23BD produced, pyruvate is only added in a 3:2 excess (compared to a 3:1 excess for formate) (Figure 4A). In this system, we started with an initial excess of formate (added as sodium formate). During the course of the reaction, we made several additions of enzymes, cofactors, or both. Our initial addition of enzymes and cofactor (24 h) increased the rate of the reaction. A subsequent addition of only cofactor (48 h) did not change the rate of reaction, suggesting that the cofactor was not limiting conversion. After that, we made three subsequent additions of both enzymes and cofactors (75, 96, and 120 h). The 75 h addition slightly increased 23BD production, while 96 and 120 h additions had no effect. The final titer of 23BD in the quad system was 1.6 ± 0.01 M (144 g/L) (Figure 4B).

The low final acetoin concentration suggests that the BDH enzyme was functional, and this is corroborated by its performance in the binary enzyme system. The final concentration of pyruvate was 290 mM, and the addition of more ALS enzyme at 120 h (along with the other three enzymes and NADH) did not allow further conversion, suggesting that the reaction was at equilibrium, although it is difficult to make strong conclusions about this in the absence of 2-acetolactate concentration data. If 2-acetolactate concentrations were high, this is likely due to the loss of activity of the ALDC enzyme, which we previously showed was the most unstable enzyme in the system (Figure 2).

In the binary system, we attempted to further increase the product titer using highly concentrated stocks of substrates. We added a bolus of the BDH enzyme and NADH at 24 and 48 h, respectively, to account for any losses in cofactor and enzyme activity due to changes in the reaction medium over time. We observed an increase in the progress of the reaction after the addition of the bolus; however, the complete reaction profile does not suggest that either the enzyme or the cofactor was limiting. We were able to readily convert 2.2 ± 0.3 M acetoin into 2.1 ± 0.2 M (189 g/L) 23BD (Figure 4C,D) which is one of the highest titers reported.^16^ Since this was more than sufficient for our purposes related to studying cell lysate systems and also due to experimental difficulties of making further increases in stock concentrations (i.e., due to limits of substrate solubility), we did not attempt to further increase titer in the binary system.

### Testing Passive pH Control Mechanism

The active pH controlled system allowed for complete conversion of molar quantities of substrates; however, there are three factors which make it less than ideal for studying cell lysates: (1) The experimental setup is cumbersome and requires a stir-plate, pH-controller/probe, and syringe pump for each experimental setup. (2) Addition of concentrated acid denatures the proteins and decreases the overall productivity of the system. (3) To accommodate the pH sensitive electrode bulb and stir bar, the minimum volume of the reaction was around 0.8 mL, which necessitated large quantities of purified enzymes (and eventually cell lysates).

To eliminate the need for active pH control, we tested whether increasing the buffer strength would be sufficient to resist changes in the pH of the system. Increasing the buffer concentration up to 500 mM slowed the increase in pH of the reaction system as compared to the 50 mM buffer (Supporting Figure S5). However, we were concerned that a 500 mM concentration of the buffer might inhibit some of the reactions when we eventually moved the system to the cell lysate. In the presence of a 50 mM buffer, we tested different concentrations of substrates in a binary system to identify the concentration at which conversion of the substrate to 23BD ceases. The reaction was set up in a 1.5 mL microfuge tube with 1 mL reaction volume, and the concentration of acetoin in different treatments varied from 10 to 1000 mM. To our surprise, we observed complete conversion of acetoin in all treatments, and the increase in pH was arrested to around a value of ∼8. (Supporting Figure S6). It was surprising since earlier (Figure 2) we did not observe complete conversion of 1000 mM acetoin to 23BD and the pH value was observed to be >9. This made us think about the role of open vs closed experimental systems. We know that one mole of CO_2_ is formed per mole of 23BD formed and in an open system the produced CO_2_ is released into the atmosphere while in the closed 1.5 mL microfuge tube CO_2_ is trapped inside.

### Reducing the Headspace to Working Volume Ratio Improves Pyruvate Conversion Efficiency

**Figure 5:**
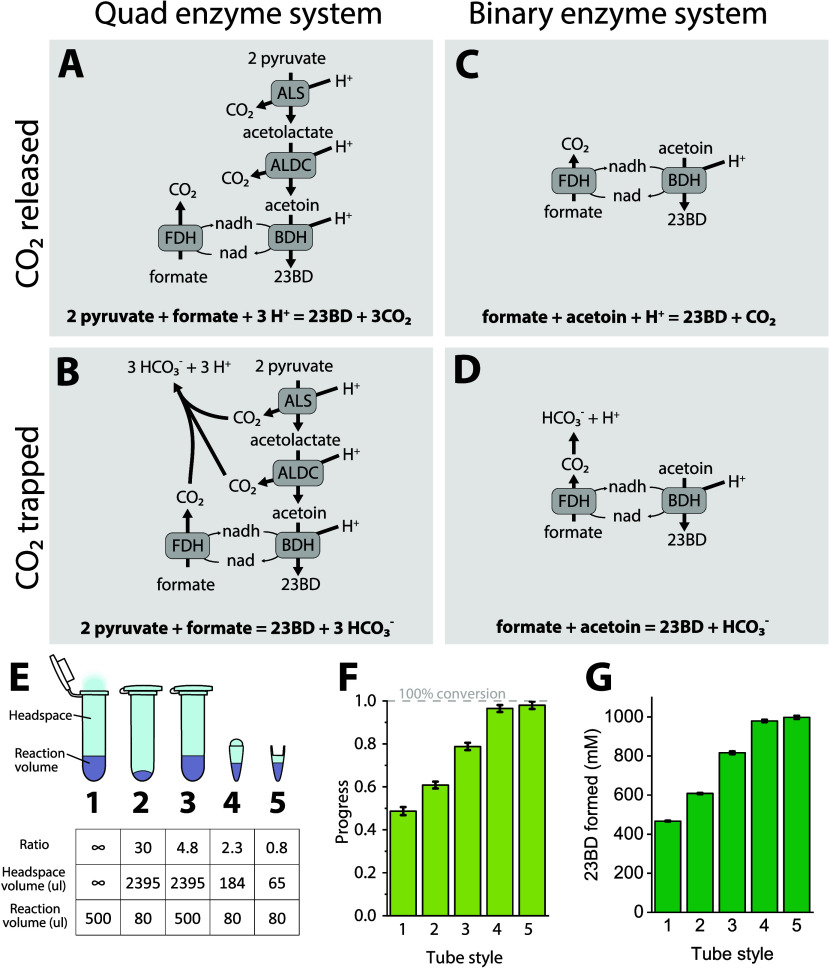
Effect of headspace volume on conversion. Panels A–D show the stoichiometry of the quad and binary enzyme systems under conditions where CO_2_ is either trapped or released. Using Cytoplasmic buffer with 50 mM HEPES. To systematically vary the effect of headspace volume, we ran the quad system in five different tube configurations, shown in Panel E. Quad enzyme system-based reactions were run in a 2 mL tube (either open or closed) or a 200 ul PCR tube with either a domed or reduced-volume cap. Reaction volumes were either 500 or 80 ul. Configurations are ordered by decreasing the headspace ratio. The initial concentration of pyruvate was 2 M. The initial concentration of formate was 1.5 M. Panel F shows the conversion of pyruvate to 23BD for each of these reaction configurations. Panel G shows the final 23BD titer. Error bars represent one standard deviation from two biological replicates.

To investigate in detail the role of open and closed systems on the conversion efficiency of pyruvate in a quad system, we tested the role of different reaction volumes and tube capacities on the conversion efficiency of the substrate (Figure 5). With an initial pH value of 7 and in the presence of 2 M pyruvate, we started the reaction in a 0.5 mL volume in a 2 mL tube under two treatments. In one treatment, CO_2_ was allowed to escape, while in another one it was trapped. At the end of 24 h, the pyruvate conversion efficiency and pH were 50% and 9.4 in the open system (Figure 5E, #1), and 80% and 8.1 in the closed system (Figure 5E, #3). Thus, preventing the release of CO_2_ from the reaction vessel can be attributed to an increase in conversion efficiency.

The results encouraged us to test whether we could further increase the conversion efficiency of pyruvate. We next monitored the conversion efficiency of pyruvate in an 80 uL reaction volume under three treatment conditions (Figure 5E, #2, #4, and #5). In #2, the reaction was allowed to proceed in a 2 mL closed tube. In #4, in a 200 uL PCR tube with normal dome caps, and in #5, in a 200 uL PCR tube with reduced volume caps (the cap protrudes into the tube, reducing the headspace volume). In treatment #2, we observed a decrease in the conversion efficiency to 60%, while in the second and third treatments, the value increased to nearly 100%. The final pH value in #2, 4, and 5 treatments was observed to be 8.6, 8.5, and 8.5, respectively. Upon analysis, we observed a clear trend in which decreasing the ratio of headspace to reaction volume increased conversion. Looking more closely at the reaction system, we also noticed that in both systems, the stoichiometry of CO_2_ production matched the stoichiometry of proton consumption (Figure 5, panels A and B). Since CO_2_ can form carbonic acid in the presence of water, we hypothesize that enhancing carbonic acid formation might allow for improved proton balance without the need for active pH control.

### Investigating the Cell Lysate System of *C. thermocellum*

Having now developed an efficient cascade reaction and a convenient experimental system that allowed high titer conversion, we set out to use the system to explore the questions that initially motivated this work: (1) is ethanol flux primarily limited by glycolysis (sugar to pyruvate) or fermentation pathways (pyruvate to ethanol), and (2) are fermentation pathways controlled primarily by carbon or electron availability.

We tested the influence of electron availability by utilizing the NADH regeneration system and observed its effect on the fate of carbon supplied either as pyruvate or as cellobiose in the cell lysate. Basically, we perturbed metabolism in cell lysates by using different permutations and combinations of pyruvate/cellobiose and formate in either the presence or absence of heterologous purified enzymes (ALS, ALDC, BDH, and FDH). Although the lysate itself contains cofactors, the concentration may be more dilute relative to what is present in the cytoplasm, and we therefore added additional cofactors to ensure that they were not limiting. We let the lysate reactions run for 24 h and then sampled to measure the substrate(s) that were consumed and the products that were formed.

**Figure 6:**
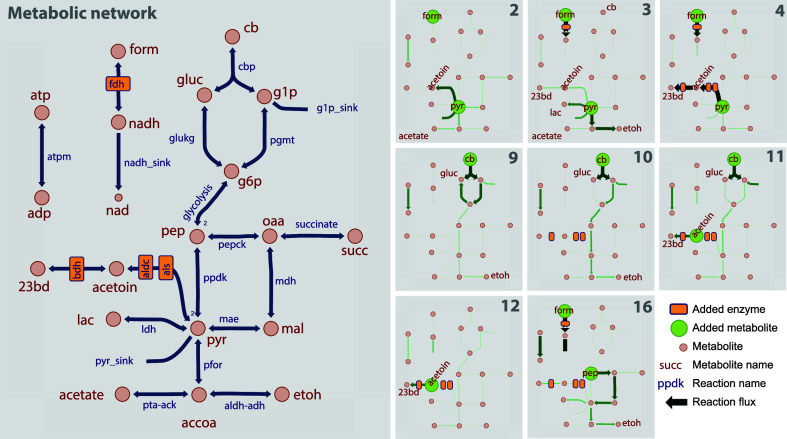
Using cascade reactions to understand cellular metabolism in lysates. The panel on the left shows a simplified network of the *C. thermocellum* central metabolism. The eight panels on the right show the effect of perturbing *C. thermocellum* lysates by adding enzymes or substrates. Fluxes calculated from the replicate 1 metabolite measurements are shown (Supporting Data set S2).

The concentrations of metabolites as determined by HPLC were used as inputs to the metabolic model to determine the carbon flux through the major pathways. Using these concentrations as boundary fluxes, we fit the data to a simple stoichiometric model (Figure 6). The degrees of freedom in this model were designed such that all of the missing carbon (i.e., that which could not be accounted for by measured products and associated CO_2_ production) would be collected in two terms: pyruvate (pyr_sink) or glucose-1-phosphate (g1p_sink), and all of the missing electrons would be collected in one term: nadh_sink. By ensuring that only one of the carbon sink reactions was available (either pyr_sink or g1p_sink, but not both), and minimizing the fluxes to that carbon sink and the nadh_sink. The stoichiometric model thus allowed us to determine the most parsimonious estimate of missing carbon and electrons that could be supported by the measured experimental data.

We performed a set of 16 experiments divided into two sets, where we included various substrates, in combination with lysate and with various coupling enzymes (Tables 1 and 2). The first set of experiments involved pyruvate as a substrate and formate for cofactor recycling (Table 1 and Figure 6). First, we tested adding only pyruvate to our cell lysate (ID 1, Supporting Data set S2). Previously, we had observed that this resulted in only small amounts of ethanol and acetate production.^6^ Here we observed a similar effect, although we now observed that about half of the pyruvate was converted to acetoin (a product we had not measured in the work of ref (6)) (Supporting Data set S2). Despite that, a large fraction of the pyruvate was still unaccounted for (i.e., high pyr_sink flux). Adding formate (Figure 6, ID 2) did not have a significant effect on fluxes, as expected, since the FDH enzyme was not present. Adding pyruvate, formate, and FDH created an NADH generation system (Figure 6, ID 3). This resulted in a very strong redirection of metabolic fluxes away from acetate and acetoin and toward lactate and ethanol. This was expected since lactate and ethanol production both consume NADH, are part of the native system for balancing excess redox equivalents, and provided evidence of the control of metabolic flux by NADH. When we added a pyruvate sink (ALS, ALDC, and BDH) in addition to the NADH source (Figure 6, ID 4), all of the pyruvate was converted to 23BD, demonstrating that our heterologous three-enzyme cascade reaction was functional in the presence of cell lysate and could out-compete the native enzymes.

**Table 1:** Reaction Setup for the Lysate Treatments Utilizing Pyruvate as a Carbon Source

	ID1	ID2	ID3	ID4	ID5	ID6	ID7	ID8
lysate	+	+	+	+	–	+	–	–
pyruvate	+	+	+	+	+	–	+	+
formate	–	+	+	+	+	+	+	+
FDH	–	–	+	+	+	+	–	–
ALS	–	–	–	+	+	+	–	–
ALDC	–	–	–	+	+	+	–	–
BDH	–	–	–	+	+	+	–	–

**Table 2:** Reaction Setup for the Lysate Treatments Utilizing Cellobiose as a Carbon Source

	ID9	ID10	ID11	ID12	ID13	ID14	ID15	ID16
CFE	+	+	+	+	+	+	+	+
cellobiose	+	+	+	–	–	–	+	–
acetoin	–	–	+	+	–	–	–	–
formate	–	–	–	–	–	–	+	+
PEP	–	–	–	–	–	–	–	+
FDH	–	–	–	–	–	–	+	+
ALS	–	+	+	+	+	–	+	+
ALDC	–	+	+	+	+	–	+	+
BDH	–	+	+	+	+	–	+	+

Our second set of tests involved adding cellobiose to the cell lysate system (Table 2, Figure 6, IDs 9–12). When only cellobiose was added to the cell lysate (Figure 6, ID 9), most of it was converted to glucose, regardless of the presence or absence of a pyruvate sink (ALS, ALDH, and BDH enzymes, ID 10). Adding an NADH sink (acetoin and BDH) redirected flux from ethanol to acetate (IDs 11 and 12), providing additional evidence that the ethanol flux is controlled by NADH levels.

A final test involved adding phosphoenolpyruvate (PEP) (Table 2, Figure 6, ID 16) along with an NADH source (formate plus FDH) and a pyruvate sink (ALS, ALDC, and BDH). The purpose of this test was to measure PEP to pyruvate conversion. Previously we had not observed any conversion of PEP in cell lysates^6^; however, the addition of the NADH generation system (FDH enzyme and formate) allowed for the conversion of about half of the initial 60 mM PEP to fermentation products (malate, succinate, lactate, ethanol, and 23BD). It is interesting that under this condition, our three-enzyme pathway for 23BD production only diverted a small amount of flux away from the native pathways (contrary to what we observed in ID 4). Further experiments will be needed to understand the cause.

In total, 16 IDs were tested (Supporting Data set S2); however, only a subset of the most informative ones are presented here in Figure 6 (however, the rest are available in Supporting Data set S2). Briefly, the conditions are (1) Pyruvate-only control. (2) Pyruvate and formate control. (3) Pyruvate, formate, and FDH. (4) Pyruvate, formate, and quad pathway. (5) No-lysate control. (6) No pyruvate control. (7) No lysate and no quad pathway control. (8) Condition 7 with the temperature decreased from 37 to 25 °C. (9) Lysate test with cellobiose. (10) Condition 9 with pyruvate sink. (11) Condition 9 with pyruvate and NADH sink. (12) No cellobiose control. (13) No substrate control. (14) Lysate-only control. (15) Condition 9 with pyruvate and NADH source. (16) Condition 15 with PEP instead of cellobiose.

## Discussion

Cell-free systems have been used to study biological systems for over a century, starting with the work of Buchner, who showed that cell-free yeast lysates could convert sugar to ethanol,^17^ resolving an important 19th century controversy about the nature of living organisms and founding the science of biochemistry.^18^ The fermentation demonstrated by Buchner was short-lived, however, achieving only about 7% of the maximum theoretical yield. Despite numerous attempts, sustained and extensive conversion of sugar to ethanol was only achieved in 1985 by Scopes and Welch, who identified ATP accumulation as the key limiting factor.^19^ Employing several different methods to avoid ATP accumulation, they were able to convert 150 mM glucose into 300 mM ethanol (i.e., 100% of theoretical yield). Recently, there has been renewed interest in using cell-free systems to prototype metabolic pathways,^11,20−22^ and to develop mathematical models of glycolysis and fermentation pathways.^23−26^

In this study, we investigated the lysate conditions that modulate the fate of pyruvate and then compared it with the flow of cellobiose carbon. We constructed a three-enzyme cascade reaction and demonstrated that it could function in cell lysates and out-compete native metabolism to provide a functional sink for pyruvate by converting it to 2,3-butanediol. We used FDH enzyme (with formate) to provide a source of NADH and BDH enzyme (with acetoin) to serve as a sink of NADH. Previously, we found that adding metabolites to lysates did not result in high levels of conversion for many metabolites.^6^ In this study, we demonstrate that in the presence of pyruvate, adding a source of NADH (FDH plus formate) led to an increase in ethanol titers which suggests that its titers are limited by NADH levels and thus cofactor recycling constraints. While in the presence of cellobiose as a carbon source, despite removing cofactor recycling constraints, we failed to observe any significant conversion of the carbon into the desired product, which suggests that additional steps upstream of pyruvate are limiting. Additional experiments will be needed to determine whether these limitations are also present in *C. thermocellum* in vivo, or whether they are due to limitations of the cell lysate system.

A key benefit of cell-free systems biology was that it allowed us to test for selective relaxation of metabolic constraints (i.e., the addition of pyruvate) that are not possible to do in living systems. Another benefit is that the use of cascade reactions for cofactor recycling and metabolite sinks is organism-agnostic and can be easily applied to novel isolates or organisms where genetic tools have not yet been developed. With the four-enzyme cascade developed in this study, we were able to develop an improved fundamental understanding of systems-level properties of the*C. thermocellum* metabolic network, as it functions in cell lysates. Our most significant finding is that carbon flux is strongly controlled by electron availability and that providing either a source or a sink for NADH dramatically reconfigures these fluxes. Although this is a well-known general principle of cellular metabolism, here we provide experimental evidence that this principle applies to the set of metabolic enzymes present in *C. thermocellum* cell lysate. Although in this work, we have mainly focused on the NAD^+^/NADH cofactor pair, the use of enzymes and appropriate cosubstrates could be extended to a variety of other cofactors, including ATP/ADP, GTP/GTP, PP_i_/P_i_, NADP^+^/NADPH, Ferredoxin (reduced/oxidized), and CoA.

We also explored the fate of cellobiose in the cell lysate system. Regardless of our experimental interventions, most of the cellobiose was converted to glucose and did not enter the glycolysis. One possible explanation for this is a stoichiometric imbalance in glycolysis. In most organisms, the energy cofactors used for the initial “pay-in” phase of glycolysis and the subsequent “pay-out” phase are the same (ATP/ADP couple). However, in *C. thermocellum*, pyrophosphate (PP_i_) is used during the “pay-in” phase,^27^ and the source of this PP_i_ is not present in the glycolysis or fermentation pathways (and indeed is not known^27−29^). Although it must function in vivo*,*^27^ the results here show that it does not function in our in vitro system. Recently we have engineered *C. thermocellum* to partially eliminate requirements for PPi,^30^ and we are interested to see if that allows increased cellobiose flux into glycolysis in vitro.

Another example of how we can use cell lysates to understand metabolism is by looking at the effect of adding different carbon substrates on the product formation. Adding cellobiose (ID 10), PEP (ID 16) or pyruvate (ID 3) with appropriate electron regeneration resulted in ethanol as the primary fermentation product. The concentration of the substrate (15 mM for cellobiose and 60 mM for PEP and pyruvate) was such that a maximum of 60 mM ethanol could have been produced in all three cases. We observed substantially more ethanol production (28 mM) with pyruvate than with either PEP (8 mM) or cellobiose (6 mM). The extent of conversion, particularly for PEP and pyruvate, is a dramatic improvement over our prior work,^6^ and we primarily attribute this to the presence of cofactor recycling systems (i.e., FDH plus formate). The large difference between product formation with PEP and product formation with pyruvate suggests a significant limitation at the PEP to pyruvate conversion. In WT *C. thermocellum*, this conversion is performed by the pyruvate phosphate dikinase (PPDK) reaction.^31^ Since the PPDK reaction requires PP_i_ as a substrate, it is possible that the lack of PEP conversion reflects a lack of PPi, possibly due to a lack of PP_i_ regeneration systems, as described above. We have previously generated strains of *C. thermocellum* where this reaction is not required^31^ and are interested to see whether those strains allow increased PEP to pyruvate conversion in vitro. An important step for subsequent research will be to understand the extent to which these phenomena observed in cell lysates are representative of *C. thermocellum* metabolism in vivo.

The unexpected appearance of acetoin in our pyruvate addition experiments highlights the utility of the cell-free systems biology approach for identifying possible routes for “missing carbon.” In many microbial fermentations, the substrate that is consumed cannot be fully accounted for in the products that are measured. This has been a recurring problem in *C. thermocellum*, where up to 30% of carbon is missing in some cases.^32^ By relaxing constraints on metabolite accumulation and allowing direct addition of intermediates, diversions of flux to unexpected products can be more systematically studied.

One interesting new contribution of this work is an improved understanding of how reaction geometry affects conversion. Although the influence of surface-area-to-volume ratios on cell-free systems is well-known,^11,33,34^ the underlying mechanism is not well understood. One common hypothesis is that a high surface area is important for oxygen transfer. There is a clear mechanistic basis for this, with reactions that have a stoichiometric requirement for oxygen. For example, the Cytomim system uses oxidative phosphorylation for energy generation,^34^ and some redox balancing systems use an oxygen-consuming NADH oxidase.^35^ Another hypothesis is that hydrophobic plastic surfaces prolong cell-free protein synthesis reactions by binding misfolded polypeptides that would otherwise inhibit the reaction.^34^ Neither of these hypotheses, however, provides suitable explanations for the current work.

The oxygen-transfer hypothesis is unlikely to explain our results for several reasons. (1) The pathways we are using do not require oxygen. (2) Limiting gas transfer *increased* conversion. (3) Cell-lysate-based experiments were performed in an anaerobic chamber due to the oxygen-sensitivity of some reactions in *C. thermocellum* metabolism. And finally, (4) for the pyruvate to 23BD reaction (which is neither oxygen-sensitive nor includes oxygen as a reactant), we observed no differences when reactions were exposed to oxygen (Figures 2, 4, 5 and Supporting Figure S6) or not (Figure 6, condition 5: no-lysate control). Another benefit of running reactions inside an anaerobic chamber is that it may promote stability of 2-acetolactate by avoiding oxygen-mediated conversion of 2-acetolactate to diacetyl.^36^

The hydrophobic plastic hypothesis^34^ is also unlikely to explain our results. The primary evidence for this is that varying the headspace composition without varying the area of the hydrophobic surface (Figure 5E, comparing configurations 1 and 3) has a strong influence on the extent of reaction progress.

Instead, we hypothesize that a proton imbalance is responsible for a lack of reaction progress in some conditions and that by trapping CO_2_, we can restore this balance, allowing increased conversion. In our system, the only sources of protons are buffer and CO_2_. The formation of 1000 mM 23BD from pyruvate requires 3000 mM protons (Figure 5B). Since a buffer can only donate protons equivalent to approximately half its concentration, only about 25 mM (less than 1%) can come from the 50 mM HEPES buffer. The rest must come from CO_2_ (i.e., via the formation of carbonic acid or other carbonate species). This is one of the primary reasons we performed these reactions with relatively high substrate concentrations: at low concentrations, the supply of protons from the buffer can obscure this effect. In fact, in a closed system, the HEPES buffer can be completely eliminated with very little effect on conversion (data not shown).

Although the properties of carbonate buffers are well-known,^37−39^ their importance in cell-free systems is not widely appreciated. In systems where CO_2_ is produced, and the demand for protons exceeds the supply, trapping CO_2_ can provide an additional source of protons. This technique worked particularly well in our system, where the stoichiometry of CO_2_ production matched proton demand (Figure 5); however, it is conceivable that in systems with different stoichiometry of CO_2_ and/or protons, releasing the CO_2_ might be more beneficial. Furthermore, the ability to trap CO_2_ in the liquid phase (via proton consumption) may be useful for reducing CO_2_ emissions associated with the production of fuels and chemicals using cell-free systems. It has potential to be used in the carbon neutral production of a compound of industrial interest, and the experimental design can be further improved to produce carbon negative production of compounds.^40^

The importance of pH control is well known, both for individual enzymes and cascade reactions^13,14,19,41^; however, the mechanism of pH change is rarely investigated. Common approaches to control pH include optimization of the initial reaction pH,^14,15,41^ increasing the buffer strength,^14^ optimizing the buffer p*K*_a_,^15^ and active pH control.^42,43^ Furthermore, since many biochemical systems are characterized in buffer systems using initial-rate measurements, effects due to proton consumption and production can often be neglected. Protons are almost universally excluded from reaction diagrams in the literature, and even some reaction databases offer confusing treatments of the topic (for example, the KEGG entry for ALS shows a reaction converting *pyruvic acid* to 2-acetolactate but inaccurately describes the compound as *pyruvate*).

Fundamentally, pH is determined by the relative concentration of protons (H^+^) and hydroxide ions (OH^–^), and thus, careful consideration of proton stoichiometry can provide insights into why pH changes as a function of reaction progress. For example, glycolysis results in a net production of 2 protons per glucose consumed, potentially reducing the pH. This may provide explanations for observed pH drops in cascade reactions involving glycolysis.^13,14,19^

Consideration of the proton stoichiometry has shed light on several other observations relevant to cascade reactions. It has been previously observed that in cell-free systems, conversion of pyruvate to 23BD is more difficult than conversion of glucose to 23BD.^11^ This is surprising since the pathway from glucose includes many additional reactions, any one of which could limit product titer. However, consideration of the proton stoichiometry suggests an alternative explanation. Conversion of one glucose molecule to two pyruvate molecules generates two protons, and these supply 2/3 of the protons required for 23BD production (if we assume excess NADH is converted back to NAD^+^ using NADH oxidase, which generates one additional proton, the reaction is completely balanced for protons). One challenge with proton accounting is finding sources for reactions with strict proton stoichiometry. For short pathways, it is advisible to check mass and charge balance by hand. We have also found the MetaCyc^44^ and BiGG^45^ databases to be reliable in this respect.

Finally, our proton accounting allowed us to identify a very simple modification to our cascade-reaction protocols that allows for increased conversion. Simply allowing CO_2_ to accumulate in closed vials is sufficient to dramatically increase the extent of conversion in the CO_2_-forming reactions.

In conclusion, we characterized a 3-enzyme pathway for conversion of pyruvate to 23BD and showed that it could perform this conversion at high titer, as long as proton balance was maintained, either by active pH control or by preventing escape of CO_2_. We used this system to perturb *C. thermocellum* lysates. We showed that the addition of cofactor recycling systems for NAD^+^/NADH allowed increased product formation from pyruvate; however, metabolites further upstream still show low conversion, possibly indicating a need for additional cofactor recycling systems (PPi, for example). We used FBA modeling to determine the magnitude of carbon and redox imbalances. Resolving these imbalances will be important to better understand the fate of carbon in these networks. Since purified enzymes and cofactor recycling systems can be added to cell lysate prepared from any organism, the approach described here provides an organism-agnostic method of studying microbial metabolism, allowing us to engineer organisms (particularly nonmodel organisms with poorly characterized physiology) more quickly.

## Materials and Methods

### Protein Purification

Enzymes used were as follows: ALS (WP_003244057.1) and ALDC (WP_017696597.1) from *Bacillus subtilis*, BDH (WP_016928044.1) from *Serratia marcescens*, and FDH (5DN9_A) from *Candida boidinii*. 6x-His-tagged nucleotide sequences of each protein were cloned into high copy number plasmids with ColE1 origin of replication derived from pET expression vectors (Agilent). His-tag were fused to the N-terminal of the Als and Bdh proteins. His-tags were fused to the C-terminal of the Aldc and Fdh proteins. Lysogeny broth (LB) medium was used in baffled flasks with either ampicillin (100 μg/mL) or kanamycin (35 μg/mL) as selection pressure. 1% of a saturated culture was used to inoculate a 1 L secondary culture in a baffled flask. This was incubated at 37 °C and allowed to reach an absorbance (OD_600_) of around 0.5. Cultures were chilled on ice for around 1 h. Isopropyl ß-D-1-thiogalactopyranoside (IPTG) was added to the chilled cultures at a final concentration of 0.3 mM to induce protein expression, and the culture was incubated for a further 16 h at 18 °C. Cells were harvested and pellets stored at −80 °C. Pellets were resuspended in lysis buffer containing 5 mM imidazole, 500 mM NaCl, 20 mM Tris-HCl (pH 7.2), 1 mg/mL lysozyme, and 1 mM of the protease inhibitor phenylmethylsulfonyl fluoride (PMSF). Cells were lysed using a microtip sonicator (Misonix S-4000, 600 W maximum output power) at 30% amplitude with 6 s on/off cycle for 20 min. The lysate was centrifuged at 4 °C and supernatant filtered using 0.45 μM PES (poly(ether sulfone)) filters. The filtrate was bound with nickel-charged affinity resin (Ni-NTA) at 4 °C on a nutator mixer for around 12 h. The bound protein was passed through a gravity column (Bio Rad), and the resin was washed with a buffer containing 15 mM imidazole, 500 mM NaCl, and 20 mM Tris-HCl (pH 7.2). Bound protein was eluted with a buffer containing 650 mM imidazole, 500 mM NaCl, and 20 mM Tris-HCl (pH 7.2). The eluted protein was dialyzed against 100 mM phosphate buffer (pH 7.0). The purity of the protein was determined by denaturing gel electrophoresis (SDS-PAGE). The bicinchoninic acid (BCA) assay (G-Biosciences) was used to estimate the protein concentration, using bovine serum albumin (BSA) as the standard. Aliquots of dialyzed proteins were snap-frozen in liquid nitrogen and stored at −80 °C. The final concentrations of proteins used in the substrate conversion assays: Als, Aldc, Bdh, and Fdh were 0.02, 0.15, 0.19, and 0.80 mg/mL, respectively. Enzyme ratios were optimized by performing pyruvate to 2,3-butanediol reactions with various concentrations of one enzyme at a time with excess of others until a maximal product titer was reached and no diacetyl or acetoin accumulation was observed. Acetolactate accumulates if the ALS step is faster than the ALDC step, and the excess acetolactate spontaneously degrades to diacetyl in the presence of oxygen. And acetoin accumulates if FDH cannot keep up with BDH activity.^36^ The approximate activities of the enzymes were as follows: ALS = 1.07 × 10^2^ U/mg protein; ALDC = 6.56 U/mg protein; BDH = 1.77 × 10^3^ U/mg protein; and FDH = 5.30 U/mg protein. Units of activity (U) are described for each enzyme assay below.

### Individual Enzyme Assays

Unless otherwise noted, enzyme assays were performed in Cytoplasmic buffer at pH 7.0 at room temperature (∼25 °C) in a 1 mL volume under anaerobic conditions. The Cytoplasmic buffer was developed to mimic *C. thermocellum* cytoplasm (Supporting Table S1). This buffer contained: 50 mM HEPES (4-(2-hydroxyethyl)-1-piperazineethanesulfonic acid), 14 mM KCl, 68 mM NaCl, 6 mM CaCl_2_, 0.03 mM MnCl_2_, 0.01 mM CoCl_2_, 0.04 mM NiCl_2_, 0.01 mM ZnSO_4_, 15 mM MgSO_4_, 5 mM NH_4_Cl, 10 mg/mL bovine serum albumin (BSA) protein, 0.4 mM thiamine pyrophosphate (TPP), 5 mM reduced glutathione (Table S2), and gave similar results to the buffer developed in Cui et al.^6^ (Supporting Figure S1).

Measurement of ALS activity (EC: 2.2.1.6) was performed using the assay of Schloss et al.^46^ The Cytoplasmic buffer was supplemented with 67 mM pyruvate. The reaction was started by the addition of ALS. Consumption of pyruvate was measured based on a decrease in absorbance 320 nm using an HP-Agilent (Model 8453) UV–vis diode array spectrophotometer. One U of activity is the amount required to form 1 umol of acetolactate (consume 2 μmole of pyruvate) under the assay conditions described above.

Measurement of the ALDC activity (EC: 4.1.1.5) was performed in two steps. First, 67 mM pyruvate was converted to ∼33 mM 2-acetolactate using 0.087 mg (19.75 U) of ALS enzyme in cytoplasmic buffer at pH 7.0. The reaction progress was measured by observing changes in absorbance at 320 nm (see the ALS assay description above). Second, we measured ALDC activity in a coupled assay with BDH. Starting with the 2-acetolactate generated in the first step, we added NADH to a final concentration of 5 mM (note that we initially used NADH at a concentration of 0.3 mM but observed high background activity relative to assay activity. These problems were reduced by increasing the NADH concentration from 0.3 to 5 mM. We therefore had to measure NADH consumption at 390 nm instead of 340 nm). This assay is described in detail in Supporting Figure S2. The purpose of the 2-step enzyme assay was to generate the 2-acetolactate (step 1) immediately prior to the coupled ALDC assay (step 2) to avoid any potential problems with 2-acetolactate instability.^36^

For measurement of BDH activity (EC: 1.1.1.B20), the Cytoplasmic buffer was supplemented with 100 mM acetoin and 0.3 mM NADH. The reaction was started by the addition of BDH, and progress was followed based on the decrease in absorbance at 340 nm (corresponding to the conversion of NADH to NAD^+^). One U of activity corresponds to the conversion of 1 μmole of NADH per minute.

For measurement of FDH activity (EC: 1.17.1.9), the Cytoplasmic buffer was supplemented with 100 mM sodium formate and 0.3 mM NAD^+^. The reaction was started by the addition of FDH, and progress was followed based on the increase in absorbance at 340 nm (corresponding to the conversion of NAD^+^ to NADH). One U of activity corresponds to the formation of 1 μmole of NADH per minute.

To measure the time-dependent decrease in enzyme activity, each protein was incubated at 37 °C, and then its activity was determined at the indicated time intervals.

### Coupled Enzyme Reactions

Unless otherwise noted, coupled enzyme assays were performed in Cytoplasmic buffer, pH 7.0 at 37 °C under anaerobic conditions. The “binary system” consisted of the BDH and FDH enzymes and mediated the conversion of formate and acetoin into CO_2_ and 2,3-butanediol. The “quad system” consisted of the ALS, ALDC, BDH, and FDH enzymes and mediated the conversion of pyruvate into CO_2_ and 2,3-butanediol. For both the binary and quad systems, concentrations of enzymes and substrates, and the reaction volume, are indicated in the figure legends. For reactions in a 2 mL tube, we used USA Scientific part number 1620–2700 with a measured internal volume of 2.47 mL. For reactions in a 0.2 mL tube, we used Applied Biosystems part 4358293. With domed caps (part 24-161A), they had a measured internal volume of 0.26 mL. With low-volume caps (part 951022089), they had a measured internal volume of 0.15 mL.

At various time points, samples were extracted from the reaction mixture. Metabolite concentrations were measured by HPLC (described below), and pH was measured using a micro pH probe (Mettler Toledo InLab Micro, 51343160).

The CO_2_ release and trapped experiments were conducted as follows: two sets of 0.5 mL reaction mixture containing tubes (capacity 2 mL) were incubated at 37 °C. One set had holes in the cap for CO_2_ release, while the other was capped to prevent CO_2_ release. To extrapolate the results to a microtube (200 uL capacity), three sets of 80uL of reaction mixture were incubated in a 2 mL tube with closed cap, a 200 uL tube with dome cap, and a 200 uL tube with reduced volume cap, respectively. The reaction was allowed to proceed, and the pH and analyte observation was recorded at 24 h.

### Fed-Batch Enzyme Assays

For pH-controlled fed-batch enzyme assays, we constructed a bioreactor (Figure S3) consisting of a 3 mL spectrophotometer cuvette (GL14 Starna Cell with septum cap) with magnetic stir bar and pH probe (Mettler Toledo InLab Micro, 51343160) that was connected to the pH controller (Hanna Instruments BL981411-0) and a syringe pump (Harvard Apparatus Model 22). Reactions were performed under aerobic conditions. Polyetheretherketone (PEEK) microtubing was used to connect the syringe to the reaction vessel. The vessel was placed on a magnetic stir plate. The pH was maintained at 7.0 using either concentrated formic acid or pyruvic acid (concentration details in the respective figure legends). The temperature was maintained at 37 °C by incubating the entire experimental apparatus in a temperature-controlled room. Fermentations were carried out in either 0.8 or 1 mL reaction volume, and samples were removed at indicated time points.

### *C. thermocellum* Lysate Assays

*C. thermocellum* strain LL1570^3^ was cultured in MTC-5 chemically defined medium with 5 g/L cellobiose^6^ in 50 mL tubes at 55 °C under anaerobic conditions. Anaerobic conditions were maintained by using a flexible vinyl anaerobic chamber (Coy Laboratory Products). At OD_600_ around 0.4, cells were harvested from 200 mL culture by centrifugation. The resulting cell pellets were washed with cytoplasmic buffer (without BSA) and finally suspended in a 250 μL final volume. Cells were lysed by the addition of 1 μL of Ready-Lyse lysozyme solution (Epicenter, WI, USA) and incubated at room temperature for 20 min. Then 1 μL of DNase I solution (Thermo Scientific, MA, USA) was added, and lysate was incubated for an additional 20 min at room temperature. The lysate was centrifuged under anaerobic conditions at 12,100 RCF for 5 min. The supernatant was harvested, and its protein concentration was determined using the BCA assay (described above). This resulting supernatant is the cell lysate that was used in subsequent assays. Cell lysate assays were performed in a 50 μL reaction volume in 200 μL PCR tubes.

### Flux-Balance Analysis

To perform flux-balance analysis (FBA), a simplified stoichiometric metabolic model of *C. thermocellum* metabolism was constructed by incorporating the reactions of glucan breakdown, glycolysis, anaplerosis, and fermentation pathways. Since we do not measure all of the possible metabolic products generated in metabolism, additional sink reactions were added for glucose-1-phosphate (g1p), pyruvate (pyr), and NADH. These reactions represent flux to unmeasured (or unknown) products. Next, net metabolite fluxes were determined based on end point measurements, subtracting initial concentrations. Exchange fluxes were allowed to vary by ±2% to account for experimental measurement errors. Flux analysis was performed using the cobrapy Python library.^47^ Fluxes were calculated by minimizing the flux to the unmeasured products, using an objective equation of g1p_sink^2^ + pyr_sink^2^ + nadh_sink^2^. Detailed analysis steps are presented in detail in a Supporting Information File (Supporting Data set S1).

### Measurement of Products

Samples for high-pressure liquid chromatography (HPLC) were treated with 2.5% trichloroacetic acid (TCA) to precipitate proteins. 0.5% sulfuric acid was added to the samples before analysis. Metabolites were estimated by Shimadzu-HPLC (Model 2030C-3D) equipped with an Aminex HPX 87H (300 × 7.8 mm) column equipped with RI and PDA detectors. The column and detector temperature was maintained at 60 °C and detector at 40 °C, respectively. 2.5 mM sulfuric acid was used as the mobile phase with a flow rate of 0.6 mL/min. Standards of each analyte were freshly prepared before analysis. Acetolactate is unstable and not available commercially, and therefore was not measured.

## Supporting Information Available

Analysis of cationic load of cell lysate; development of the cytoplasmic buffer; *C. thermocellum* strain LL1570 cultivated in MTC-5; development of an assay to determine ALDC activity; custom-built pH-controlled enzyme reaction vessel; influence of co-substrate feed on conversion of pyruvate to 2,3-butanediol; influence of buffer pH and ionic strength on substrate conversion to 23BD in the quad enzyme system; and influence of varying concentrations of acetoin and formate on pH and efficiency of 2,3-butanediol formation (PDF)
Flux modeling supplemental data (XLSX)
cth_lystate_flux_analysis dataset (ZIP)

## References

[ref1] Lynd L. R.; Beckham G. T.; Guss A. M.; Jayakody L. N.; Karp E. M.; Maranas C.; McCormick R. L.; Amador-Noguez D.; Bomble Y. J.; Davison B. H.; Foster C.; Himmel M. E.; Holwerda E. K.; Laser M. S.; Ng C. Y.; Olson D. G.; Román-Leshkov Y.; Trinh C. T.; Tuskan G. A.; Upadhayay V.; Vardon D. R.; Wang L.; Wyman C. E. Toward Low-Cost Biological and Hybrid Biological/catalytic Conversion of Cellulosic Biomass to Fuels. Energy Environ. Sci. 2022, 15 (3), 938–990. 10.1039/D1EE02540F.

[ref2] Mazzoli R.; Olson D. G. G. Clostridium Thermocellum: A Microbial Platform for High-Value Chemical Production from Lignocellulose. In Advances in Applied Microbiology; Intergovernmental Panel on Climate Change, Ed.; Elsevier Inc.: Cambridge, 2020; pp 111–161. 10.1016/bs.aambs.2020.07.004 32948265

[ref3] Hon S.; Holwerda E. K.; Worthen R. S.; Maloney M. I.; Tian L.; Cui J.; Lin P. P.; Lynd L. R.; Olson D. G. Expressing the *Thermoanaerobacterium Saccharolyticum pforA* in Engineered *Clostridium Thermocellum* Improves Ethanol Production. Biotechnol. Biofuels 2018, 11 (1), 242 10.1186/s13068-018-1245-2. 30202437 PMC6125887

[ref4] Tian L.; Papanek B.; Olson D. G.; Rydzak T.; Holwerda E. K.; Zheng T.; Zhou J.; Maloney M.; Jiang N.; Giannone R. J.; Hettich R. L.; Guss A. M.; Lynd L. R. Simultaneous Achievement of High Ethanol Yield and Titer in *Clostridium Thermocellum*. Biotechnol. Biofuels 2016, 9 (1), 116 10.1186/s13068-016-0528-8. 27257435 PMC4890492

[ref5] Jacobson T. B.; Korosh T. K.; Stevenson D. M.; Foster C.; Maranas C.; Olson D. G.; Lynd L. R.; Amador-Noguez D. In Vivo Thermodynamic Analysis of Glycolysis in Clostridium Thermocellum and Thermoanaerobacterium Saccharolyticum Using 13 C and 2 H Tracers. mSystems 2020, 5 (2), 84 10.1128/mSystems.00736-19. PMC7380578 32184362

[ref6] Cui J.; Stevenson D.; Korosh T.; Amador-Noguez D.; Olson D. G.; Lynd L. R. Developing a Cell-Free Extract Reaction (CFER) System in Clostridium Thermocellum to Identify Metabolic Limitations to Ethanol Production. Front. Energy Res. 2020, 8 (June), 72 10.3389/fenrg.2020.00072.

[ref7] Cornish-Bowden A. Fundamentals of Enzyme Kinetics; Elsevier: 2014.

[ref8] Zhang L.; Sun J.; Hao Y.; Zhu J.; Chu J.; Wei D.; Shen Y. Microbial Production of 2,3-Butanediol by a Surfactant (serrawettin)-Deficient Mutant of Serratia Marcescens H30. Journal of Industrial Microbiology & Biotechnology. 2010, 37, 857–862. 10.1007/s10295-010-0733-6. 20467779

[ref9] Ma C.; Wang A.; Qin J.; Li L.; Ai X.; Jiang T.; Tang H.; Xu P. Enhanced 2,3-Butanediol Production by Klebsiella Pneumoniae SDM. Appl. Microbiol. Biotechnol. 2009, 82 (1), 49–57. 10.1007/s00253-008-1732-7. 18949476

[ref10] Kim J.-W.; Kim J.; Seo S.-O.; Kim K. H.; Jin Y.-S.; Seo J.-H. Enhanced Production of 2,3-Butanediol by Engineered Saccharomyces Cerevisiae through Fine-Tuning of Pyruvate Decarboxylase and NADH Oxidase Activities. Biotechnology for Biofuels. 2016, 10.1186/s13068-016-0677-9. PMC5148919 27990176

[ref11] Kay J. E.; Jewett M. C. Lysate of Engineered Escherichia Coli Supports High-Level Conversion of Glucose to 2,3-Butanediol. Metab. Eng. 2015, 32, 133–142. 10.1016/j.ymben.2015.09.015. 26428449

[ref12] Zhang L.; Xu Q.; Zhan S.; Li Y.; Lin H.; Sun S.; Sha L.; Hu K.; Guan X.; Shen Y. A New NAD(H)-Dependent Meso-2,3-Butanediol Dehydrogenase from an Industrially Potential Strain Serratia Marcescens H30. Appl. Microbiol. Biotechnol. 2014, 98 (3), 1175–1184. 10.1007/s00253-013-4959-x. 23666479

[ref13] Caschera F.; Noireaux V. Synthesis of 2.3 Mg/mL of Protein with an All Escherichia Coli Cell-Free Transcription–translation System. Biochimie 2014, 99, 162–168. 10.1016/j.biochi.2013.11.025. 24326247

[ref14] Karim A. S.; Rasor B. J.; Jewett M. C. Enhancing Control of Cell-Free Metabolism through pH Modulation. Synth. Biol. 2020, 5 (1), ysz027 10.1093/synbio/ysz027.

[ref15] Jewett M. C.; Swartz J. R. Mimicking the Escherichia Coli Cytoplasmic Environment Activates Long-Lived and Efficient Cell-Free Protein Synthesis. Biotechnol. Bioeng. 2004, 86 (1), 19–26. 10.1002/bit.20026. 15007837

[ref16] Straathof A. J. J. Transformation of Biomass into Commodity Chemicals Using Enzymes or Cells. Chem. Rev. 2014, 114 (3), 1871–1908. 10.1021/cr400309c. 23987659

[ref17] Buchner E.; Rapp R. Alcoholic Fermentation without Yeast Cells. Ber. Dtsch. Chem. Ges. 1901, 34 (2), 1523–1530. 10.1002/cber.19010340231.

[ref18] Kohler R. The Background to Eduard Buchner’s Discovery of Cell-Free Fermentation. J. Hist. Biol. 1971, 4 (1), 35–61. 10.1007/BF00356976. 11609437

[ref19] Welch P.; Scopes R. K. Studies on Cell-Free Metabolism: Ethanol Production by a Yeast Glycolytic System Reconstituted from Purified Enzymes. J. Biotechnol. 1985, 2 (5), 257–273. 10.1016/0168-1656(85)90029-X.

[ref20] Dudley Q. M.; Anderson K. C.; Jewett M. C. Cell-Free Mixing of Escherichia Coli Crude Extracts to Prototype and Rationally Engineer High-Titer Mevalonate Synthesis. ACS Synth. Biol. 2016, 5, 1578 10.1021/acssynbio.6b00154. 27476989 PMC6728267

[ref21] Dudley Q. M.; Karim A. S.; Jewett M. C. Cell-Free Metabolic Engineering: Biomanufacturing beyond the Cell. Biotechnol. J. 2015, 10, 69–82. 10.1002/biot.201400330. 25319678 PMC4314355

[ref22] Krüger A.; Mueller A. P.; Rybnicky G. A.; Engle N. L.; Yang Z. K.; Tschaplinski T. J.; Simpson S. D.; Köpke M.; Jewett M. C. Development of a Clostridia-Based Cell-Free System for Prototyping Genetic Parts and Metabolic Pathways. Metab. Eng. 2020, 62, 95–105. 10.1016/j.ymben.2020.06.004. 32540392

[ref23] Bujara M.; Schümperli M.; Pellaux R.; Heinemann M.; Panke S. Optimization of a Blueprint for in Vitro Glycolysis by Metabolic Real-Time Analysis. Nat. Chem. Biol. 2011, 7 (5), 271–277. 10.1038/nchembio.541. 21423171

[ref24] Hold C.; Billerbeck S.; Panke S. Forward Design of a Complex Enzyme Cascade Reaction. Nat. Commun. 2016, 7, 1–8. 10.1038/ncomms12971. PMC5052792 27677244

[ref25] Smallbone K.; Messiha H. L.; Carroll K. M.; Winder C. L.; Malys N.; Dunn W. B.; Murabito E.; Swainston N.; Dada J. O.; Khan F.; Pir P.; Simeonidis E.; Spasić I.; Wishart J.; Weichart D.; Hayes N. W.; Jameson D.; Broomhead D. S.; Oliver S. G.; Gaskell S. J.; McCarthy J. E. G.; Paton N. W.; Westerhoff H. V.; Kell D. B.; Mendes P. A Model of Yeast Glycolysis Based on a Consistent Kinetic Characterisation of All Its Enzymes. FEBS Lett. 2013, 587 (17), 2832–2841. 10.1016/j.febslet.2013.06.043. 23831062 PMC3764422

[ref26] Teusink B.; Passarge J.; Reijenga C. A.; Esgalhado E.; Van Der Weijden C. C.; Schepper M.; Walsh M. C.; Bakker B. M.; Van Dam K.; Westerhoff H. V.; Snoep J. L. Can Yeast Glycolysis Be Understood in Terms of in Vitro Kinetics of the Constituent Enzymes? Testing Biochemistry. Eur. J. Biochem. 2000, 267 (17), 5313–5329. 10.1046/j.1432-1327.2000.01527.x. 10951190

[ref27] Zhou J.; Olson D. G.; Argyros D. A.; Deng Y.; van Gulik W. M.; van Dijken J. P.; Lynd L. R. Atypical Glycolysis in *Clostridium Thermocellum*. Appl. Environ. Microbiol. 2013, 79 (9), 3000–3008. 10.1128/AEM.04037-12. 23435896 PMC3623140

[ref28] Schroeder W. L.; Kuil T.; van Maris A. J. A.; Olson D. G.; Lynd L. R.; Maranas C. D. A Detailed Genome-Scale Metabolic Model of Clostridium Thermocellum Investigates Sources of Pyrophosphate for Driving Glycolysis. Metab. Eng. 2023, 77, 306–322. 10.1016/j.ymben.2023.04.003. 37085141

[ref29] Kuil T.; Hon S.; Yayo J.; Foster C.; Ravagnan G.; Maranas C. D.; Lynd L. R.; Olson D. G.; van Maris A. J. A. Functional Analysis of H + -Pumping Membrane-Bound Pyrophosphatase, ADP-Glucose Synthase, and Pyruvate Phosphate Dikinase as Pyrophosphate Sources in Clostridium Thermocellum. Appl. Environ. Microbiol. 2021, 88, e0185721 10.1128/aem.01857-21. 34936842 PMC8863071

[ref30] Hon S.; Jacobson T.; Stevenson D. M.; Maloney M. I.; Giannone R. J.; Hettich R. L.; Amador-Noguez D.; Olson D. G.; Lynd L. R. Increasing the Thermodynamic Driving Force of the Phosphofructokinase Reaction in Clostridium Thermocellum. Appl. Environ. Microbiol. 2022, 88, e0125822 10.1128/aem.01258-22. 36286488 PMC9680637

[ref31] Olson D. G.; Hörl M.; Fuhrer T.; Cui J.; Zhou J.; Maloney M. I.; Amador-Noguez D.; Tian L.; Sauer U.; Lynd L. R. Glycolysis without Pyruvate Kinase in Clostridium Thermocellum. Metab. Eng. 2017, 39, 169–180. 10.1016/j.ymben.2016.11.011. 27914869

[ref32] Ellis L. D.; Holwerda E. K.; Hogsett D.; Rogers S.; Shao X.; Tschaplinski T.; Thorne P.; Lynd L. R. Closing the Carbon Balance for Fermentation by Clostridium Thermocellum (ATCC 27405). Bioresour. Technol. 2012, 103 (1), 293–299. 10.1016/j.biortech.2011.09.128. 22055095

[ref33] Jewett M. C.; Calhoun K. A.; Voloshin A.; Wuu J. J.; Swartz J. R. An Integrated Cell-Free Metabolic Platform for Protein Production and Synthetic Biology. Mol. Syst. Biol. 2008, 4, 220 10.1038/msb.2008.57. 18854819 PMC2583083

[ref34] Voloshin A. M.; Swartz J. R. Efficient and Scalable Method for Scaling up Cell Free Protein Synthesis in Batch Mode. Biotechnol. Bioeng. 2005, 91 (4), 516–521. 10.1002/bit.20528. 15937883

[ref35] Opgenorth P. H.; Korman T. P.; Bowie J. U. A Synthetic Biochemistry Molecular Purge Valve Module That Maintains Redox Balance. Nat. Commun. 2014, 5 (May), 4113 10.1038/ncomms5113. 24936528

[ref36] Kobayashi K.; Kusaka K.; Takahashi T.; Sato K. Method for the Simultaneous Assay of Diacetyl and Acetoin in the Presence of Alpha-Acetolactate: Application in Determining the Kinetic Parameters for the Decomposition of Alpha-Acetolactate. J. Biosci. Bioeng. 2005, 99 (5), 502–507. 10.1263/jbb.99.502. 16233823

[ref37] Kernohan J. C. The pH-Activity Curve of Bovine Carbonic Anhydrase and Its Relationship to the Inhibition of the Enzyme by Anions. Biochimica et Biophysica Acta (BBA) - Enzymology and Biological Oxidation 1965, 96 (2), 304–317. 10.1016/0926-6593(65)90014-7. 14298834

[ref38] Cao L.; Caldeira K.; Jain A. K. Effects of Carbon Dioxide and Climate Change on Ocean Acidification and Carbonate Mineral Saturation. Geophys. Res. Lett. 2007, 34, 028605 10.1029/2006GL028605.

[ref39] Mitchell M. J.; Jensen O. E.; Cliffe K. A.; Maroto-Valer M. M. A Model of Carbon Dioxide Dissolution and Mineral Carbonation Kinetics. Proceedings of the Royal Society A: Mathematical, Physical and Engineering Sciences 2010, 466 (2117), 1265–1290. 10.1098/rspa.2009.0349.

[ref40] Liew F. E.; Nogle R.; Abdalla T.; Rasor B. J.; Canter C.; Jensen R. O.; Wang L.; Strutz J.; Chirania P.; De Tissera S.; Mueller A. P.; Ruan Z.; Gao A.; Tran L.; Engle N. L.; Bromley J. C.; Daniell J.; Conrado R.; Tschaplinski T. J.; Giannone R. J.; Hettich R. L.; Karim A. S.; Simpson S. D.; Brown S. D.; Leang C.; Jewett M. C.; Köpke M. Carbon-Negative Production of Acetone and Isopropanol by Gas Fermentation at Industrial Pilot Scale. Nat. Biotechnol. 2022, 40 (3), 335–344. 10.1038/s41587-021-01195-w. 35190685

[ref41] Alberto Alcalá-Orozco E.; Grote V.; Fiebig T.; Klamt S.; Reichl U.; Rexer T. A Cell-Free Multi-Enzyme Cascade Reaction for the Synthesis of CDP-Glycerol. Chembiochem 2023, 24 (21), e202300463 10.1002/cbic.202300463. 37578628

[ref42] Sherkhanov S.; Korman T. P.; Chan S.; Faham S.; Liu H.; Sawaya M. R.; Hsu W.-T.; Vikram E.; Cheng T.; Bowie J. U. Isobutanol Production Freed from Biological Limits Using Synthetic Biochemistry. Nat. Commun. 2020, 11 (1), 4292 10.1038/s41467-020-18124-1. 32855421 PMC7453195

[ref43] Neuhauser W.; Steininger M.; Haltrich D.; Kulbe K. D.; Nidetzky B. A pH-Controlled Fed-Batch Process Can Overcome Inhibition by Formate in NADH-Dependent Enzymatic Reductions Using Formate Dehydrogenase-Catalyzed Coenzyme Regeneration. Biotechnol. Bioeng. 1998, 60, 277–282. 10.1002/(sici)1097-0290(19981105)60:3<277::aid-bit2>3.0.co;2-e. 10099429

[ref44] Caspi R.; Billington R.; Keseler I. M.; Kothari A.; Krummenacker M.; Midford P. E.; Ong W. K.; Paley S.; Subhraveti P.; Karp P. D. The MetaCyc Database of Metabolic Pathways and Enzymes - a 2019 Update. Nucleic Acids Res. 2020, 48 (D1), D445–D453. 10.1093/nar/gkz862. 31586394 PMC6943030

[ref45] King Z. A.; Lu J.; Dräger A.; Miller P.; Federowicz S.; Lerman J. A.; Ebrahim A.; Palsson B. O.; Lewis N. E. BiGG Models: A Platform for Integrating, Standardizing and Sharing Genome-Scale Models. Nucleic Acids Res. 2016, 44 (D1), D515–D522. 10.1093/nar/gkv1049. 26476456 PMC4702785

[ref46] Schloss J. V.; Van Dyk D. E.; Vasta J. F.; Kutny R. M. Purification and Properties of Salmonella Typhimurium Acetolactate Synthase Isozyme II from Escherichia Coli HB101/pDU9. Biochemistry 1985, 24 (18), 4952–4959. 10.1021/bi00339a034. 3907697

[ref47] Ebrahim A.; Lerman J. A.; Palsson B. O.; Hyduke D. R. COBRApy: COnstraints-Based Reconstruction and Analysis for Python. BMC Syst. Biol. 2013, 7, 74 10.1186/1752-0509-7-74. 23927696 PMC3751080

