# Supplementary material for: Cell-Free Systems Biology: Characterizing Central Metabolism of Clostridium thermocellum with a Three-Enzyme Cascade Reaction
Source: ACS Synth Biol. 2024 Oct 10;13(11):3587–99. doi: 10.1021/acssynbio.4c00405 (PMC11574923; doi:10.1021/acssynbio.4c00405)
Supplement: Supplementary file 1 — sb4c00405_si_001.pdf [file sb4c00405_si_001.pdf]

Supporting Information

Cell-Free Systems Biology: Characterizing Central Metabolism of *Clostridium thermocellum* with a Three-Enzyme Cascade Reaction

Authors

S. Bilal Jilani[1], Markus Alahuhta[2], Yannick J. Bomble[2], Daniel G. Olson[1*]

Affiliations

[1] Thayer School of Engineering at Dartmouth College, Hanover, NH 03755
[2] National Renewable Energy Laboratory, Biosciences Center, Golden, CO 80401
* To whom correspondence should be addressed: daniel.g.olson@dartmouth.edu

Corresponding author information

Daniel G. Olson, Thayer School of Engineering at Dartmouth College, Hanover, NH 03755, daniel.g.olson@dartmouth.edu

Key Words

2,3-butanediol; acetolactate synthase; acetolactate decarboxylase; 2,3-butanediol dehydrogenase; formate dehydrogenase; *Hungateiclostridium*; *Ruminiclostridium*; *Acetivibrio thermocellus*

Supporting Dataset S1. A zip file archive containing the following files
me23escher_v2.yml: a YML file with python dependencies required for the analysis
cth_lystate_flux_analysis_12-8-2023.ipynb: a JupyterNotebook file with the code used to perform the stoichiometric modeling as well as descriptions of each step.
cth_simple_lysate_model.csv: a computer-readable text based description of the stoichiometric model
Lysate data_Dan DO.xlsx: input metabolite concentration data from HPLC measurements
cth_compact_flux_map1.json: an Escher metabolic map used for visualization of fluxes
cth_more_compact_map_v2.json: an Escher metabolic map used for visualization of fluxes

Supporting Dataset S2. An Excel file with experimental conditions, metabolite measurements, and flux modeling outputs.
Flux modeling supplemental data.xlsx

Table S1. Analysis of cationic load of cell lysate. Wild Type *C. thermocellum* LL1004 was cultivated in MTC-5 medium (5g/L cellobiose) and grown to mid-log phase (OD_{600} ~0.4 to 0.6). Cells were harvested and washed twice with high purity water (Ultratrace analysis grade) and resuspended in the same. Cells were lysed using a microtip sonicator. The lysate was centrifuged and the ion concentrations in the supernatant were measured by ICP-MS. These ion concentrations were used to constitute the cytoplasmic buffer, listed below.

**Property**	**Units**	**Value in cytoplasm (standard deviation)**	**Value in cytoplasmic buffer**	**Notes**
pH	unitless		7.0	
Cl^-	mM	not measured	99.2	
S	mM	16.0 (5.6)	15	Added as SO_4^{2-} ion in buffer
N	mM	not measured	5	Added as NH^{4+} ion in buffer
Mg^{2+}	mM	14.6 (0.7)	15	
K^+	mM	14.3 (7.3)	14	
Ca^{2+}	mM	5.5 (0.4)	6	
Mn^{2+}	mM	0.03 (0.01)	0.03	
Co^{2+}	mM	0.01 (0.00)	0.01	
Ni^{2+}	mM	0.04 (0.04)	0.04	
Cu^{2+}	mM	0.01 (0.00)	0.01	
Zn^{2+}	mM	0.01 (0.01)	0.01	
Na^{2+}	mM	67.6 (76.6)	68	

Table S2. Development of the Cytoplasmic buffer. The ion concentrations obtained via ICP-MS of cell lysate were used to constitute the cytoplasmic buffer for experiments.

**Component**	**Concentration** (mM unless indicated)
HEPES (pH=7.0)	50
KCl	14
NaCl	68
$CaCl_2$	6
$MnCl_2$	0.03
$CoCl_2$	0.01
$NiCl_2$	0.04
$CuCl_2$	0.01
$ZnSO_4$	0.01
$MgSO_4$	15
NH_4Cl	5
Bovine serum albumin (BSA)	10 mg/mL
Thiamine pyrophosphate (TPP)	0.4
Reduced-glutathione	5

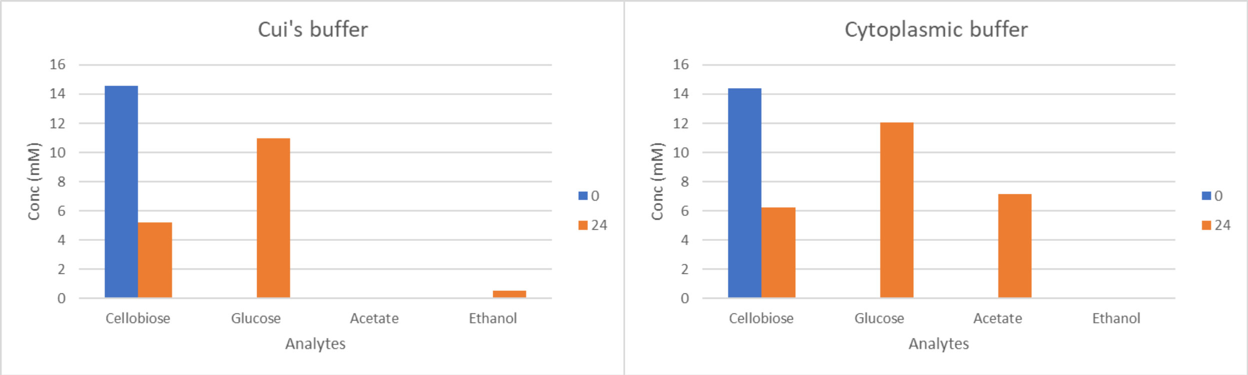

Figure S1. *C. thermocellum* strain LL1570 [3] was cultivated in MTC-5 (5 g/L cellobiose) medium until mid-exponential phase (OD_{600} ~0.4 to 0.6). Cells were harvested and washed twice with Lysis buffer with following composition: 50 mM Tris-HCl (pH=7.0), 5 mM $MgCl_2$, 60 mM KCl, 50 mM $NaHCO_3$, 5 mM reduced glutathione, 0.4 mM TPP, 5 mM NH_4Cl and finally cells were suspended in the lysis buffer. Cells were lysed by adding lysozyme and DNAase and centrifuged. The total protein content of the cell lysate supernatant was determined by BCA. Cell lysate at a final concentration of 3 mg/mL was used in reaction mixes labeled as Cui's buffer [6] and Cytoplasmic buffer. Composition of cytoplasmic buffer is as listed in Table S2 and of Cui's buffer is as follows: 1 mM each of ATP, GTP, PPi, NAD^+, $NADP^+$ and Coenzyme A, 0.1 mg/mL of BSA. The concentration of cellobiose in each reaction mix was 5 g/L. The incubation temperature was 37°C. Both buffers allow for similar consumption of cellobiose. The cytoplasmic buffer allows increased 2-carbon products (ethanol + acetate) compared to the Cui et al. buffer. Blue bars indicate initial conditions. Orange bars represent results after 24 h of incubation.

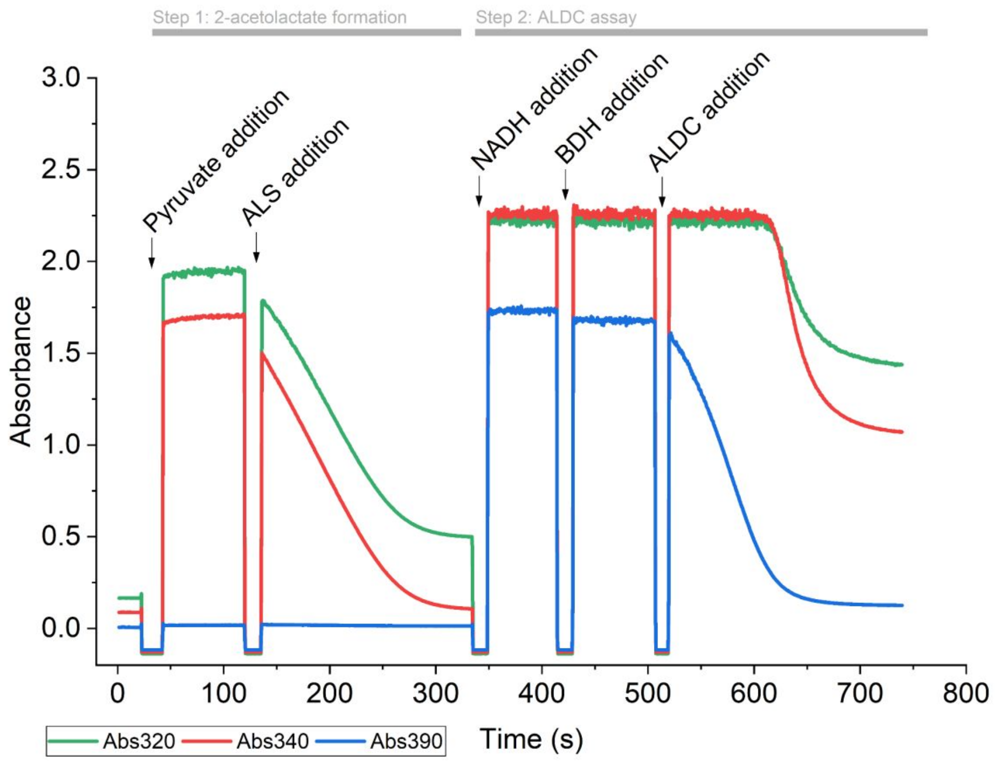

Figure S2. Development of an assay to determine ALDC activity. In Step #1 (0-350 seconds), 66.7 mM of pyruvate was added to cytoplasmic buffer (pH=7.0). After stabilization of signal at 320 nm, (corresponding to pyruvate), 40 uL of ALS was added for conversion of pyruvate to acetolactate, measured by a decrease in absorbance at 320 nm. In Step #2 (350-370 seconds), the acetolactate generated in step 1 was converted to acetoin (i.e. ALDC activity), and this was measured using BDH as a reporter enzyme. Reaction progress was followed by measuring the concentration using an absorbance signal at 390 nm. The 390 nm wavelength was chosen over the 340 nm wavelength commonly used for NADH measurements because the extinction coefficient is lower at 390 nm, allowing for higher concentrations of NADH to be used. 5 mM NADH was used. The assay was performed at room temperature (25°C).

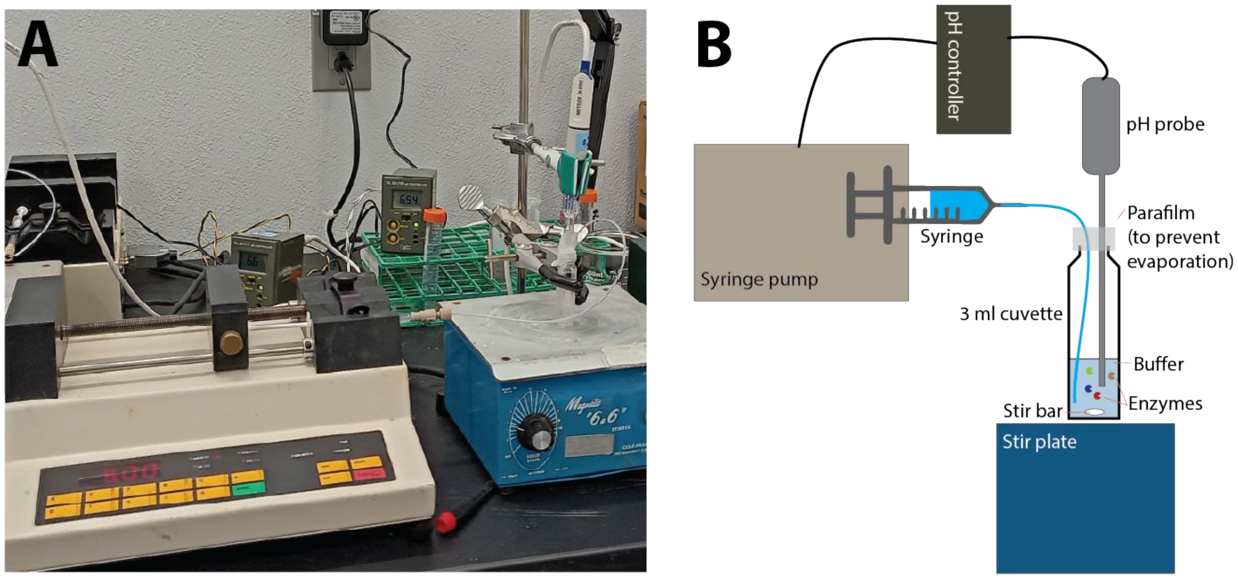

Figure S3. Custom-built pH-controlled enzyme reaction vessel. (A) Photograph of experimental setup. (B) Schematic diagram of the experimental setup. A quartz cuvette was used as a reaction vessel with a stir-bar on a magnetic stirrer. The pH of the system was maintained by using a pH probe connected to a syringe pump via a pH controller, which enabled automated addition of either pyruvic or formic acid. The temperature was maintained at 37°C by placing the complete setup in a temperature controlled warm room.

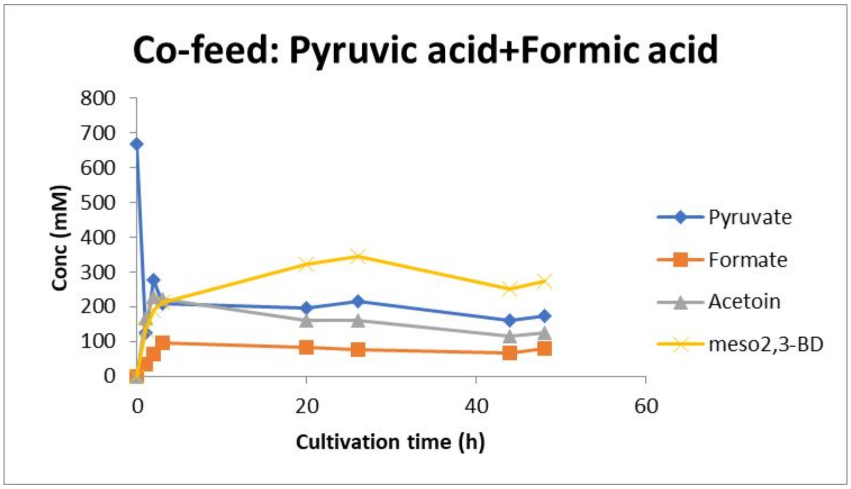

Figure S4. The influence of co-substrate feed on conversion of pyruvate to 2,3-butanediol. The initial reaction mixture contained 100 mM formate and 1 M pyruvate. The pH was maintained at pH 7.0 using a co-feed of 14.1 M pyruvic acid and 7.05 M formic acid in a 0.8 mL reaction mixture.

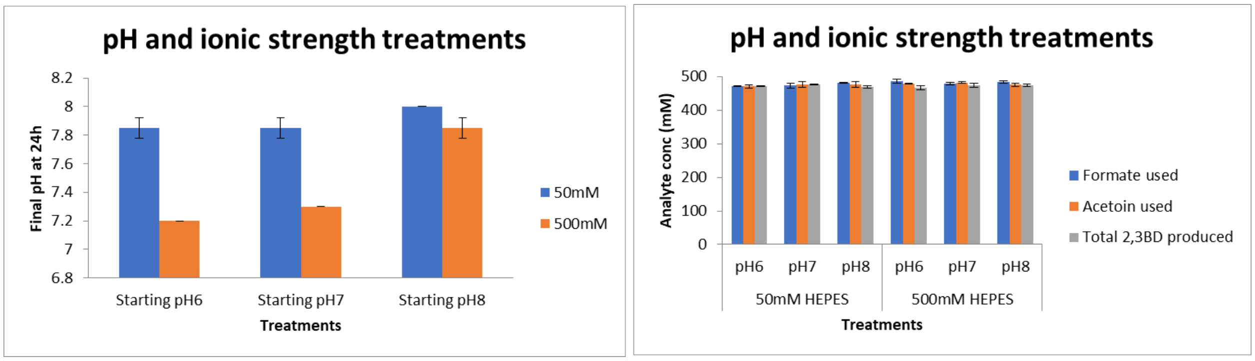

Figure S5. The influence of buffer pH and ionic strength on substrate conversion to 23BD in the quad enzyme system. A 1 mL reaction mixture was set up in a closed 1.5 mL tube (A) The reaction mixture contained either 50 mM or 500 mM HEPES buffer and starting pH at either 6, 7 or 8. After 24 h incubation at 37°C the final pH in all 50 mM HEPES buffer tubes was ~8, while in 500 mM HEPES buffer, the tubes with starting pH values of 6 and 7 showed lower increases in pH. This suggests that the higher ionic strength buffer is successful in controlling the increase of pH of the system. (B) The substrate conversion and product formation was comparable across different pH and ionic strength treatments.

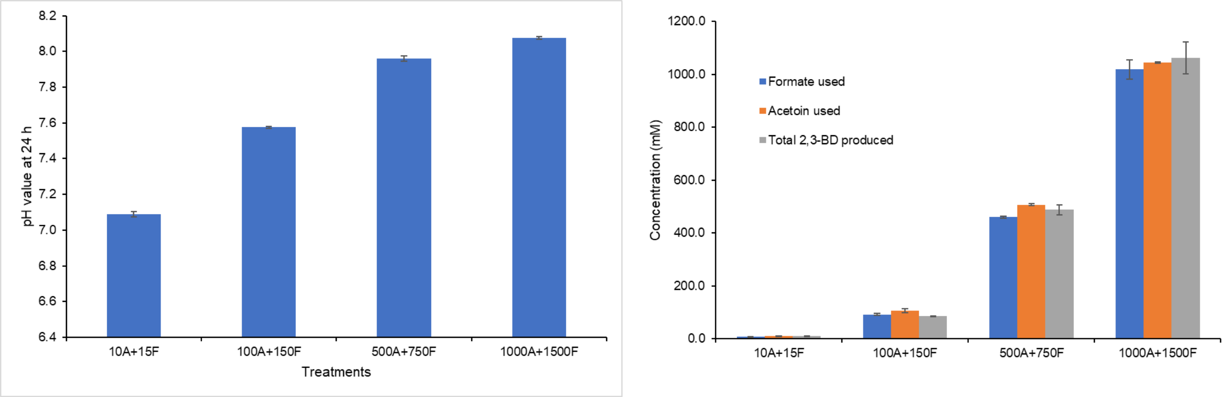

Figure S6.The influence of varying concentrations of acetoin and formate on pH and efficiency of 2,3-butanediol formation. 1mL reaction volume with starting pH=7.0 in a closed 1.5mL tube was started and readings recorded at 24h. X-axis denote the concentrations of acetoin (A) and formate (F) in mM. (A) Final pH observed in each tube (B) Concentrations of respective substrate consumed and product formed at the end of the reaction.
